# Recent Advances in the Use of Diorganyl Diselenides as Versatile Catalysts

**DOI:** 10.3390/molecules28186614

**Published:** 2023-09-14

**Authors:** Gabriel Pereira da Costa, Gustavo Bierhals Blödorn, Angelita Manke Barcellos, Diego Alves

**Affiliations:** 1 Laboratório de Síntese Orgânica Limpa (LASOL), Centro de Ciências Químicas, Farmacêuticas e de Alimentos (CCQFA), Universidade Federal de Pelotas (UFPel), Pelotas 96010-900, Brazil; gustavoblodorn@hotmail.com; 2 Escola de Química e Alimentos, Universidade Federal do Rio Grande (FURG), Rio Grande 96203-900, Brazil

**Keywords:** catalysts, diorganyl diselenide, organic synthesis

## Abstract

The importance of organoselenium compounds has been increasing in synthetic chemistry. These reagents are well-known as electrophiles and nucleophiles in many organic transformations, and in recent years, their functionality as catalysts has also been largely explored. The interest in organoselenium-based catalysts is due to their high efficacy, mild reaction conditions, strong functional compatibility, and great selectivity. Allied to organoselenium catalysts, the use of inorganic and organic oxidants that act by regenerating the catalytic species for the reaction pathway is common. Here, we provide a comprehensive review of the last five years of organic transformations promoted by diorganyl diselenide as a selenium-based catalyst. This report is divided into four sections: (1) cyclisation reactions, (2) addition reactions and oxidative functionalisation, (3) oxidation and reduction reactions, and (4) reactions involving phosphorus-containing starting materials.

## 1. Introduction

**Figure 1:**
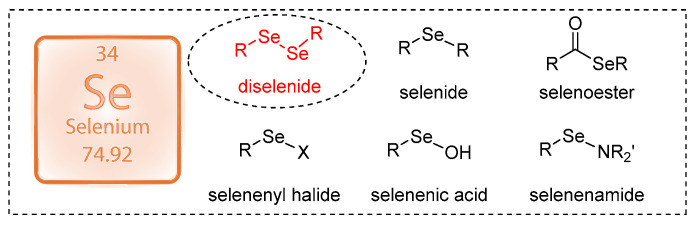
Structure of a few selenium compounds.

Selenium is an essential trace element in diets, discovered in the 18th century by the Swedish chemist Jöns Jacob Berzelius. Although the toxic properties of selenium compounds have been reported, this is a metabolisable element that does not accumulate in organisms [1,2,3,4]. Nowadays, organoselenium compounds play an important role in different fields, such as medicinal chemistry, organic synthesis, and materials science. The biological and medicinal interests in organoselenium compounds are mainly related to their antioxidant, anti-inflammatory, anticancer, and antimicrobial properties, among other properties [5,6,7]. The interest of organic chemists was generated by the discovery of the selenoxide elimination reaction in the early 1970s. Since then, an extraordinary number of different reactions have been developed once selenium reagents showed their versatility to be applied as electrophiles and nucleophiles. Therefore, a considerable number of divalent and stable species of selenium compounds have been described in the literature. Among these, we can highlight the selenides, diselenides, and selenoesters (Figure 1) [8,9,10,11,12,13].

Organoselenium reagents reached a new dimension through their application as organocatalysts. The utility of these reagents has shown unique properties, such as their high selectivity, mild working conditions, and largely transition metal-free reactions that avoid harmful toxic waste. Many new compounds have been prepared in selenium-catalysed reactions, demonstrating their efficiency in oxidation reactions (epoxidation, dihydroxylation, among others) [14], C–Z (Z = O, N, halogen, SCF_3_) bond formation [15], and cyclisation [16], among other organic reactions [17,18,19,20,21,22].

In the past decade, organoselenium chemistry has been extensively reviewed in the literature, focused on its synthesis and biological applications, as well as its use as intermediates and catalysts in organic transformations [23,24,25,26,27]. However, the use of diorganyl diselenides as catalysts has not been considered in the literature as a separate subject and is poorly detailed. In this sense, to improve the understanding of the application of diorganyl diselenide as a catalyst, the review was divided into four sections: (1) cyclisation reactions, (2) addition and oxidative functionalisation reactions, (3) oxidation and reduction reactions, and (4) reactions involving starting materials containing phosphorous. Furthermore, the compounds were numbered using numbers and letters (e.g., **1a**, **1b**; **2a**, **3a**, etc.). In cases where a certain class of compounds has more than 26 examples, a second letter was used after the number (e.g., **1z**, **1aa**, **1ab**, **1ac**, etc.). Roman numerals were used to indicate intermediates in the reaction mechanisms, and capital letters were used for the selenium catalysts.

## 2. Application of Diorganyl Diselenide as a Catalyst

### 2.1. Cyclisation Reactions

In this section, various cyclisation reactions catalysed by diorganyl diselenide will be discussed. Most of the reactions involve substrates bearing amide or amine functional groups, generating heterocyclic compounds. The reaction conditions for these reactions are diverse, carried out by conventional heating, under light irradiation, and through electrochemistry, showing the potential of diorganyl diselenide catalysts. Among the various diorganyl diselenides used to promote the synthesis of the target products, only diaryl diselenides were utilised. Diphenyl diselenide was the most common catalyst among the protocols, which were operationally simple and proceeded under mild reaction conditions. In addition, for the reactions involving regio-, diastereo-, and enantioselectivity cyclisation, it was found that the use of diaryl diselenides containing chiral moiety was necessary. Furthermore, in this section, it can be noted that the use of dialkyl diselenides, such as dibenzyl, was not efficient.

**Scheme 1:**
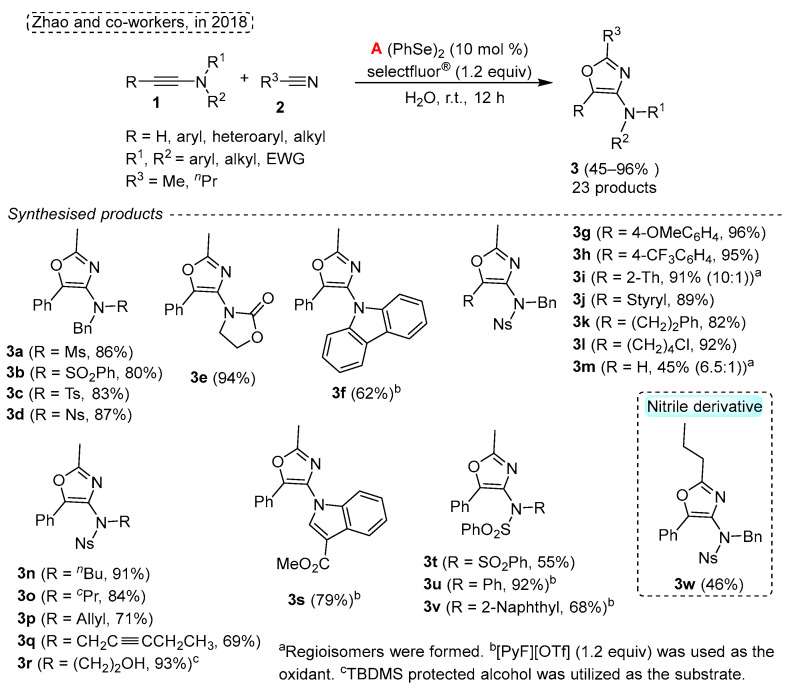
Synthesis of polysubstituted oxazoles **3** [28].

Zhao and co-workers [28], in 2018, adapted the conditions previously optimised (synthesis of ynone **29** described in this review, Section 2.2) for the synthesis of polysubstituted oxazoles **3** reacting internal alkynes **1** with H_2_O and acetonitrile **2**, which act as the solvent and nitrogen source. The reactions were performed in the presence of 10 mol% of diphenyl diselenide **A** as a catalyst over a stoichiometric amount of Selectfluor^®^ (1.2 equiv.) as an oxidant (and fluorine source to form the electrophilic selenium species) at room temperature (r.t.) for 12 h (Scheme 1). These conditions were efficiently applied to several ynamides **1**, containing both aryl and alkyl substituents in the alkynyl or *N*-position, which reacted with acetonitrile **2**. In these cases, a wide range of desired oxazoles **3** (23 products) were prepared in yields ranging from moderate to excellent (45–96%). Moreover, butyronitrile can be used as a solvent; in this case, oxazole **3w** substituted with propyl portion in the C2-position was obtained at a 46% yield (Scheme 1).

**Scheme 2:**
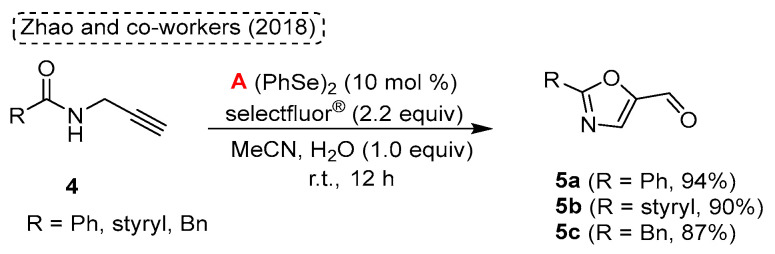
Synthesis of oxazole aldehydes **5** [28].

When an increase in the amount of Selectfluor^®^, from 1.2 equiv. to 2.2 equiv., in the conditions described above was tested, *N*-propargylamides **4** could be used as starting materials to obtain oxazole aldehydes **5** in excellent yields (87–94%). The 2-substituted oxazole **5** with phenyl **5a**, styryl **5b,** and benzyl **5c** groups were obtained in yields of 94%, 90%, and 87%, respectively (Scheme 2). Despite the good yields, only three compounds were synthesised using this methodology.

**Scheme 3:**
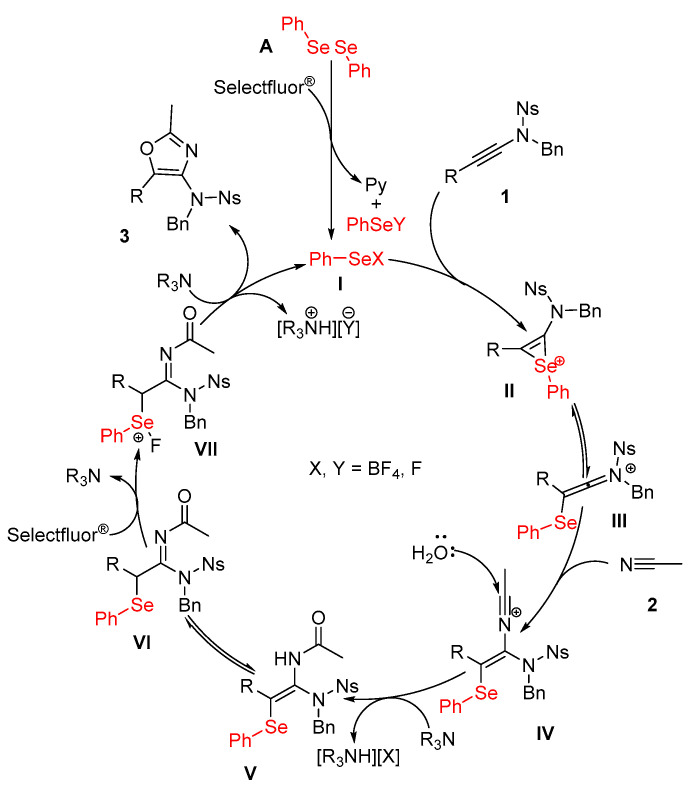
Mechanism proposed for the synthesis of oxazole **3**.

The mechanism proposed by the authors starts with the formation of electrophilic selenium species **I** through the reaction of diphenyl diselenide **A** with Selectfluor^®^. Electrophilic intermediate **I** reacts with ynamide **1** to form seleniranium **II**, which is in equilibrium with stable keteniminium ion **III**. Afterwards, acetonitrile **2** reacts with intermediate **III**, followed by hydrolysis to give intermediate **V**. Thereafter, the isomerisation of intermediate **V** leads to **VI**, which, in the presence of Selectfluor^®^, can be oxidised to produce intermediate **VII**. Finally, the target oxazole **3** is formed by the elimination of the selenium group of intermediate **VII** by attacking the carbonyl group, followed by isomerisation (Scheme 3).

**Scheme 4:**
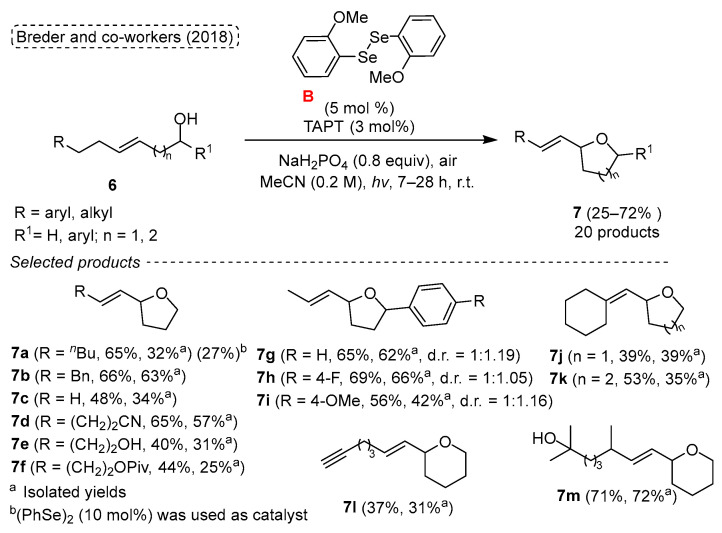
Synthesis of cyclic ethers **7** by intramolecular reaction [29].

Previous reports described using selenium-*π*-acids as efficient catalysts to promote the reaction of alkenes with carboxylic acids or hydrogen phosphates under aerobic conditions. In this regard, Breder and his research group [29] described the new organocatalytic and visible light protocol for the synthesis of cyclic ethers **7** (intramolecular reaction). In this new organocatalytic approach, a light-induced electron transfer process was applied in the reaction with alcohol **6** by using an aerobic dehydrogenative allylic etherification reaction, utilising ambient air as the terminal oxidant (Scheme 4).

The best conditions for the aerobic cycloetherification of unsaturated alcohols **6** was established when these starting materials were reacted in the presence of 5 mol% of bis(2-methoxyphenyl) diselenide **B** as a catalyst, 3 mol% of 2,4,6-tris(4-anisyl)pyrylium tetrafluoroborate (TAPT), 0.8 equiv. of NaH_2_PO_4_, atmospheric air, MeCN (0.2 M) as a solvent, and *hν* [light emitting diode (LED) irradiation at 465 nm] for 7–28 h at r.t. Under these conditions, the targets tetrahydrofuran and tetrahydropyran **7** (20 compounds) were obtained in yields ranging from low to good (25–72%). This method tolerates several starting materials **6** functionalised with nitrile, ester, halogen, carbonate, ether, imide, and unprotected hydroxy groups, as well as primary and secondary alcohols (Scheme 4).

**Scheme 5:**
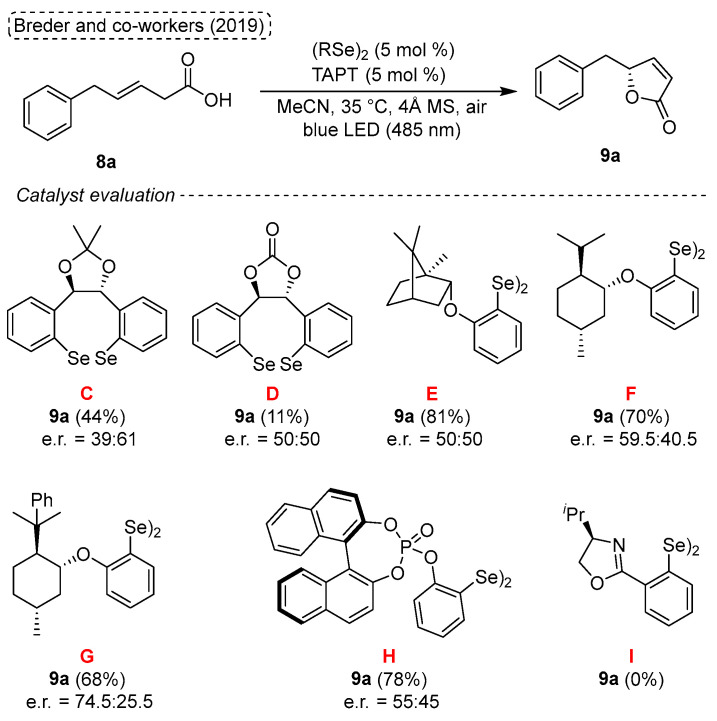
Catalyst evaluation for the synthesis of **9a** [30].

Breder and co-workers [30], in 2019, described the use of cyclic and noncyclic chiral selenium-*π*-acid catalysts, such as the derivatives of (−)-menthol **F**, (−)-8-phenylmenthol **G**, (−)-borneol **E,** and (*R*)-BINOL **H**, in the enantioselective aerobic intramolecular lactonisation of 5-phenylpent-3-enoic acid **8a**. This starting material was reacted in the presence of 10 mol% of diselenides and 5 mol% of TAPT, using acetonitrile as the solvent under atmospheric air and 465 nm irradiation for 20 h. When carbonate **D**- or acetal **C**-protected 1,2-diol derivatives of the cyclic diselenide were tested, low and moderate yields as well as a poor enantiomeric ratios (e.r.) for compound **9a** were obtained: 11% (50:50) and 44% (39:61), respectively. The diselenide derivatives of (−)-borneol **E** and (*R*)-BINOL **H** were efficiently applied, yielding the intramolecular product **9a** at 81% and 78%, respectively, but with a low e.r. On the other hand, when the diselenide derivatives of (−)-menthol **F** and (−)-8-phenylmenthol **G** were tested, lactonisation product **9a** was formed with good yields (70% and 68%, respectively). In these cases, the catalyst **F** derivative of (−)-menthol gave a lower e.r. (59.5:40.5) when compared to the catalyst **G** derivative of (−)-8-phenylmenthol. Additionally, the diselenide containing chiral oxazole derivative **I** was checked in this reaction. The authors suggested that catalyst **I** suffered degradation under photoredox conditions, and the reaction did not occur. Despite the high number of different diorganyl diselenides studied as a catalyst, the authors did not explore the expansion nor the limitations of the method of other starting materials (Scheme 5).

**Scheme 6:**
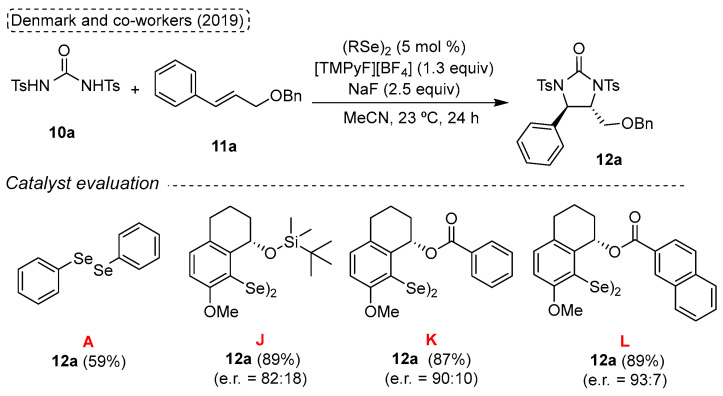
Catalyst evaluation for the synthesis of **12a** [31].

In 2019, Denmark and co-workers described [31] the use of chiral and enantioenriched diselenides **J**–**L** as catalysts in the reaction of *N*,*N*′-bistosyl urea **10** with simple alkene **11** to synthesise imidazolidin-2-one **12** by using the first intermolecular enantioselective *syn*-diamination protocol. The authors performed a study on the reaction of (*E*)-(3-(benzyloxy)prop-1-en-1-yl)benzene **11a** with *N*,*N*′-bistosylurea **10a** with different diselenides species, **A, J**–**L**, in amounts of 5 mol%, 1.3 equiv. of 2,4,6-trimethylpyrylium tetrafluoroborate [TMPyF][BF_4_], 2.5 equiv. of NaF, and MeCN as the solvent at 23 °C for 24 h (Scheme 6).

A moderate yield (59%) of target 1,2-ditosyl imidazolidin-2-one **12a** was obtained when diphenyl diselenide **A** was used as a catalyst; however, in this case, no e.r. was reported by the authors. Subsequently, chiral diselenide catalysts **J**–**L** were evaluated, and in all cases, the yield and e.r. increased. Similar yields for **12a** were obtained when using the catalyst substituted with *O*-TBDMS (*^t^*butyldimethylsilyl)-protected group **J**, benzoate ester **K,** and 2-naphthoate ester **L** (89%, 87%, and 89%, respectively). Moreover, catalysts **J**–**L** demonstrated good e.r. but had better results when the 2-naphthoate ester **L** derivative was used as catalyst (an e.r. of **12a** = 93:7). The chiral diselenides induced excellent selectivity in the products; however, they are not readily available and need a few steps for their preparation (Scheme 6).

**Scheme 7:**
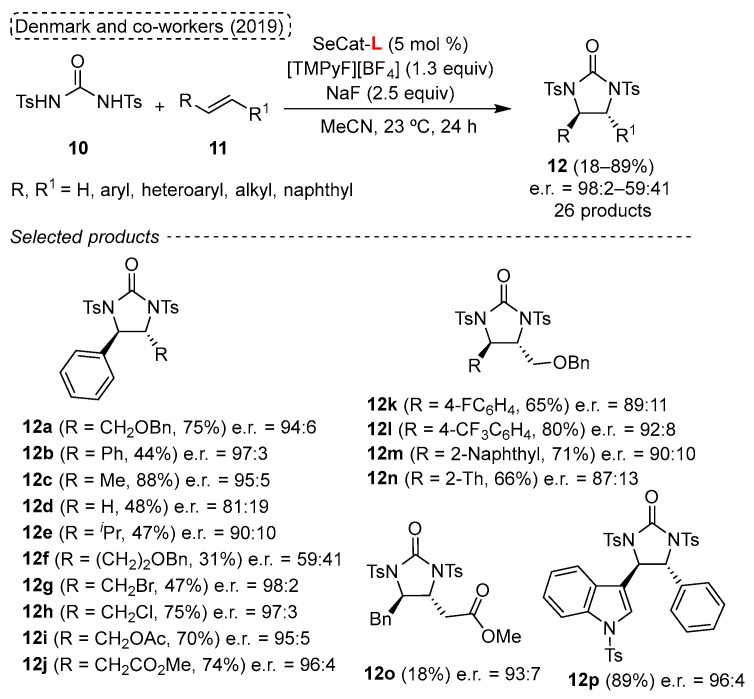
Synthesis of 1,2-ditosyl imidazolidin-2-ones **12** [31].

The standard conditions were efficiently applied to diaryl, aryl-alkyl, and dialkyl alkenes **11** in a reaction with *N*,*N*′-bis tosyl urea **10**, obtaining a wide range of 1,2-ditosyl imidazolidine-2-ones **12** (26 products). In general, the yields of the **12** compounds ranged from moderate to excellent (44–89%), except for compounds **12f** and **12o,** which were obtained with lower yields. Compound **12o** was formed at 18% with a high e.r. (93:7), whereas compound **12f** was isolated at a 31% yield but showed a significant decrease in e.r. (59:41) (Scheme 7).

**Scheme 8:**
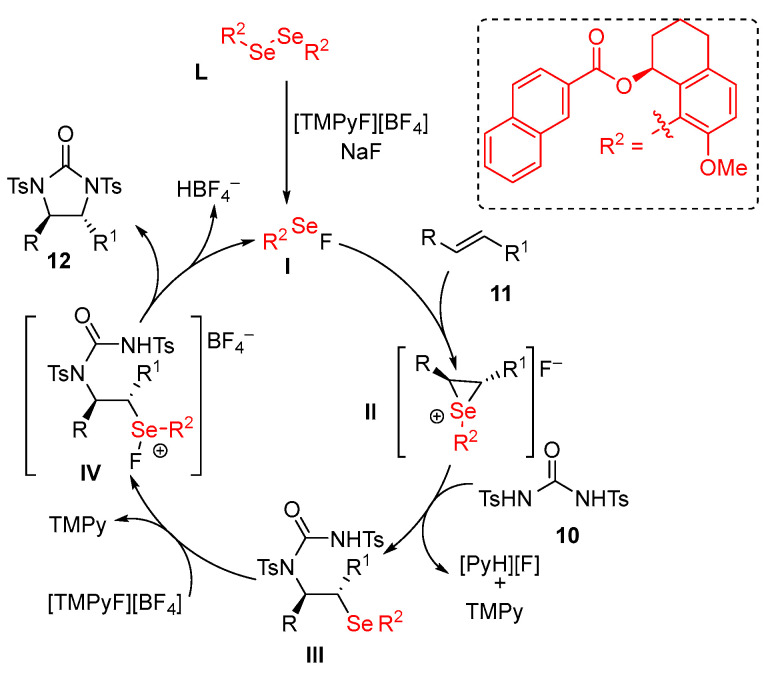
Mechanism proposed for the synthesis of 1,2-ditosyl imidazolidin-2-one **12**.

The proposed mechanism starts with the formation of the electrophilic arylselenium species **I** through the reaction of diselenide **L** with [TMPyF][BF_4_]. Intermediate **I** reacts with alkene **11** to produce seleniranium ion intermediate **II** in a concerted step, which is opened by *N*,*N*′-bistosyl urea **10**, yielding intermediate **III**. Another equivalent of [TMPyF][BF_4_] reacts with the intermediate **III** that was previously formed to give intermediate **IV**. Finally, intermediate **IV** suffers an intramolecular reaction to form target product **12** and regenerate electrophilic arylselenium **I** in a new cycle (Scheme 8).

**Scheme 9:**
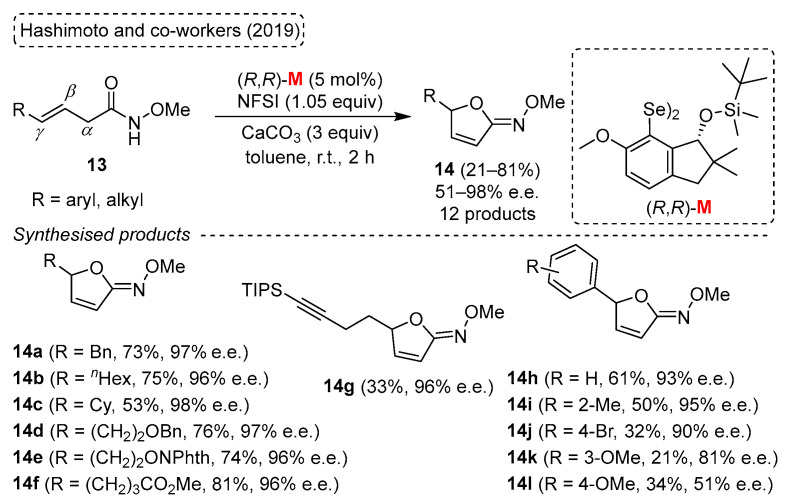
Synthesis of iminolactone **14** [32].

Moreover, in 2019, Hashimoto and co-workers [32] described the synthesis of diselenide **M**, as well as its application as a chiral selenium *π*-acid catalyst in the iminolactonisation of *N*-methoxy *β*,*γ*-unsaturated amide **13**, with high enantioselectivity induced by chiral diorganyl diselenide **M** (Scheme 9). In this approach, *β*,*γ*-unsaturated amide **13** is reacted in the presence of 5 mol% of diselenide **M** as a catalyst (3 equiv. of CaCO_3_, 1.05 equiv. of *N*-fluorobenzenesulfonimide (NFSI) in dry toluene) at r.t. under an argon atmosphere. After 2 h under these conditions, the target iminolactone **14** (12 compounds) was obtained in yields ranging from low to excellent (21–81%) and an enantiomeric excess (e.e.) ranging from 51% to 98%. In general, the protocol tolerated a broad range of starting materials **13**, containing alkyl and aryl substituents in the *β* position of *β*,*γ*-unsaturated amide **13**, as well as some products replaced with phthalimide (Phth) and triisopropyl silane (TIPS) groups. Additionally, methyl ester portions were obtained, which are of great importance since they are functionalisable compounds and can be used as building blocks for future reactions. Still, the tailored catalyst gave a high enantioselectivity for the products synthesised (generally above 90% for e.e.), except for the compounds **14l** and **14k** (51% and 81% e.e., respectively) (Scheme 9).

**Scheme 10:**
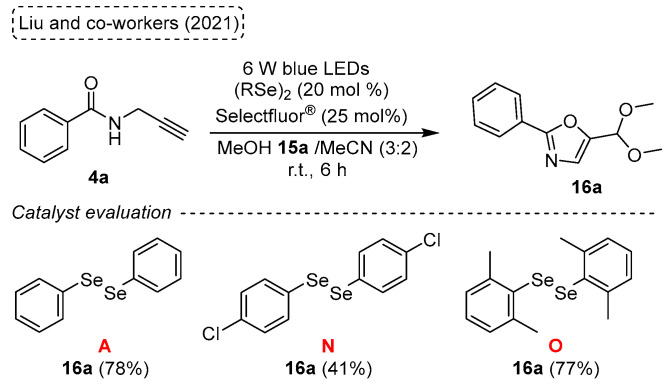
Catalyst evaluation for the synthesis of oxazole acetal **16a** [33].

In 2021, Liu and co-workers [33] reported the use of diselenides, Selectfluor^®^, and visible light as a catalytic system to synthesise oxazole acetal **16** from a one-pot reaction of *N*-propargylamide **4** with alcohol **15**. In this approach, besides the catalytic amount of diselenides and Selectfluor^®^, blue LEDs were also used to catalyse the aerobic oxidation, using air as the terminal oxidant and promoting the cyclisation step, followed by aromatisation, and finally leading to the acetalisation of product **16**, forming three new carbon-oxygen bonds (Scheme 10). 

Initially, different diaryl diselenides (in amounts of 20 mol%) were evaluated in the reaction using *N*-(prop-2-yn-1-yl)benzamide **4a** in the presence of Selectfluor^®^ (25 mol%) and 6 W blue LEDs, with MeOH **15a** and MeCN (3:2) as a solvent mixture. Under these conditions at r.t. for 6 h, 5-(dimethoxymethyl)-2-phenyloxazole **16a** was obtained at 78% and 77% when using diphenyl diselenide **A** and bis(2,6-dimethylphenyl) diselenide **O**, respectively. With bis(4-chlorophenyl) diselenide **N** as the catalyst, a significant decrease in the yield (41%) of target product **16a** was observed. Likely due to its relatively simple synthetic and ready commercial availability, diphenyl diselenide **A** was chosen as the ideal catalyst to study (Scheme 10).

**Scheme 11:**
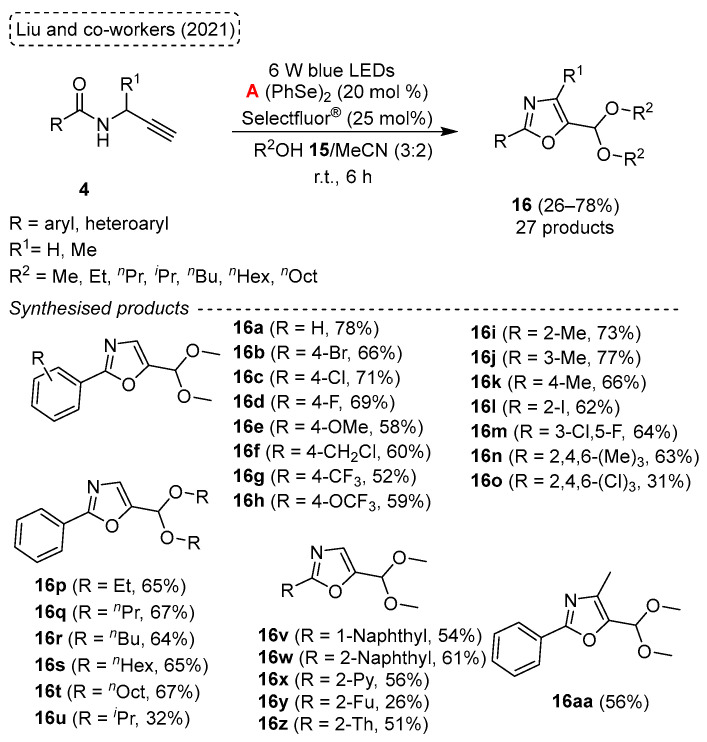
Synthesis of oxazole acetals **16** [33].

After the best reaction conditions were established, the authors investigated different *N*-propargylamides, **4**, substituted with aryl (containing electron-withdrawing groups (EWGs) and electron-donating groups (EDGs)), naphthyl (1- or 2-substituted), and heteroaryl (Py, Fu, and Th) groups bonded to the amide portion to give the desired product **16** (27 products) in yields ranging from 26% to 78%. Afterwards, alcohol **15** was also evaluated, changing methanol to EtOH (**16p**, 65%), *^n^*PrOH (**16q**, 67%), *^i^*PrOH (**16u**, 32%), *^n^*BuOH (**16r**, 64%), *^n^*HexOH (**16s**, 65%), and *^n^*OctOH (**16t**, 67%); the yields of products of **16** were slightly lower in most cases. The method was extended to *N*-(but-3-yn-2-yl)benzamide, which gave the target product **16aa** in a moderate yield of 56% (Scheme 11). In addition to the wide range of synthesised compounds, the method shows the advantage of not using a photocatalyst and utilising a smaller amount of Selectfluor^®^ compared to the work described earlier in this review.

Mechanistic studies were performed, such as carrying out the reaction in the dark and under anaerobic conditions (Ar atmosphere), as well as in the presence of radical scavengers 2,2,6,6-tetramethyl-1-piperidinyloxy (TEMPO) and 2,6-ditertbutyl-4-methylphenol (BHT). All cases showed a marked decrease in the yield, suggesting a radical pathway as well as stressing the importance of oxygen. Controlled experiments using vinyl selenium species **III** were also performed. This possible intermediate **III** was reacted under standard conditions, affording a good yield of the target product **16** (70%); still, intermediate **III** can be transformed into oxazole aldehyde **5** (another possible intermediate).

**Scheme 12:**
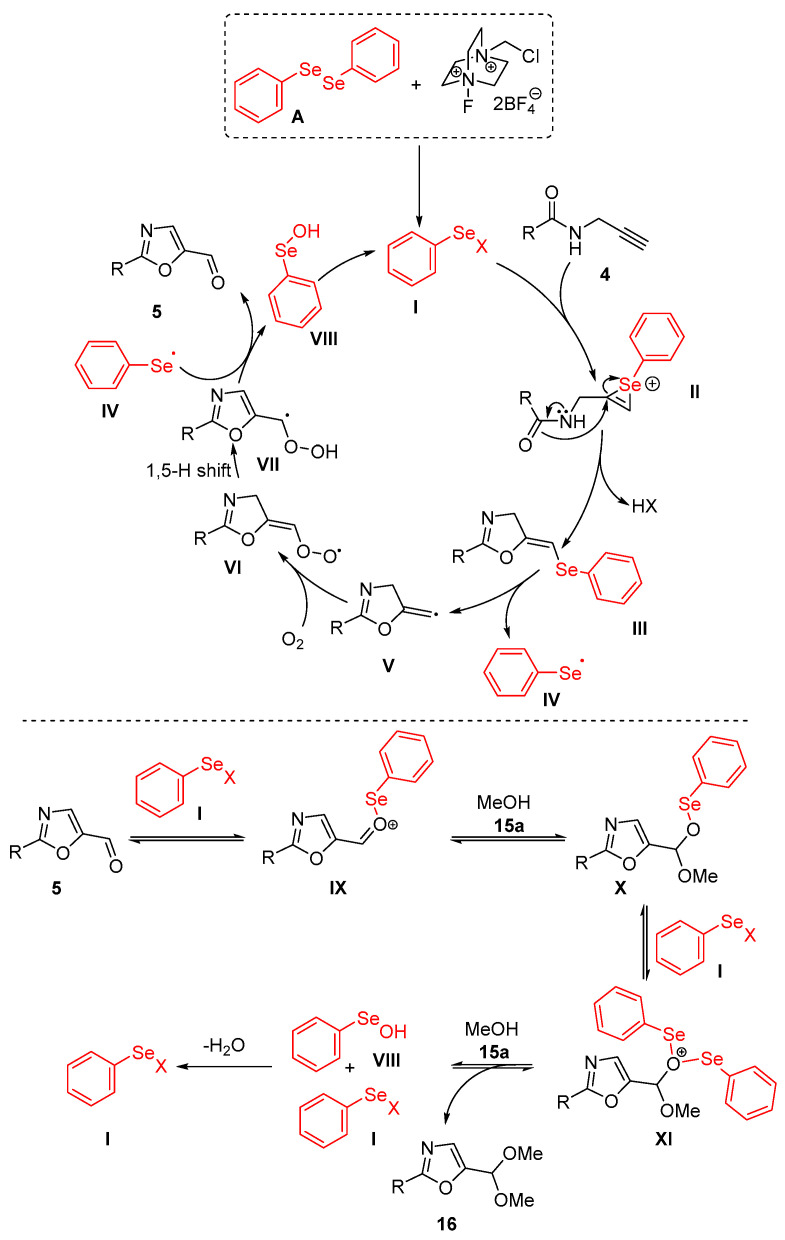
Mechanism proposed for the synthesis of oxazole acetal **16**.

After some mechanistic studies and based on previous reports in the literature, a plausible mechanism was described by the authors. The first step is the formation of Se-electrophile species **I** through the reaction of diselenides **A** with Selectfluor^®^. The intermediate reacts with *N*-propargylamide **4** to perform the electrophile cyclisation (**II**) to obtain intermediate **III**. The visible light acts on intermediate **III** in a homolytic cleavage C–Se bond, forming the PhSe and vinyl radical species **IV** and **V**, respectively. Afterwards, the peroxy-radical species **VI** is formed through the capture of O_2_ by the vinyl radical **V**. The **VI** previously formed suffers a 1,5-hydrogen migration, yielding intermediate **VII**, which reacts with PhSe radical species **IV** to form oxazole aldehyde **5** and benzene selenium acid **VIII** (which regenerate Se-electrophile species **I** after treatment with HF or HBF_4_) (Scheme 12).

In parallel, Se-electrophile species **I** can act as a Lewis acid (Se···O interaction), activating the carbonyl group of oxazole aldehyde **5** to perform an acetalisation reaction with alcohol **15** to form hemiacetal **X**. Hemiacetal **X** reacts with another Se-electrophile species, **I**, to form the intermediate **XI**, which, in the presence of alcohol **15**, gives the desired product, **16** (ketal), and regenerates Se-electrophile species **I** to a new catalytic cycle (Scheme 12).

**Scheme 13:**
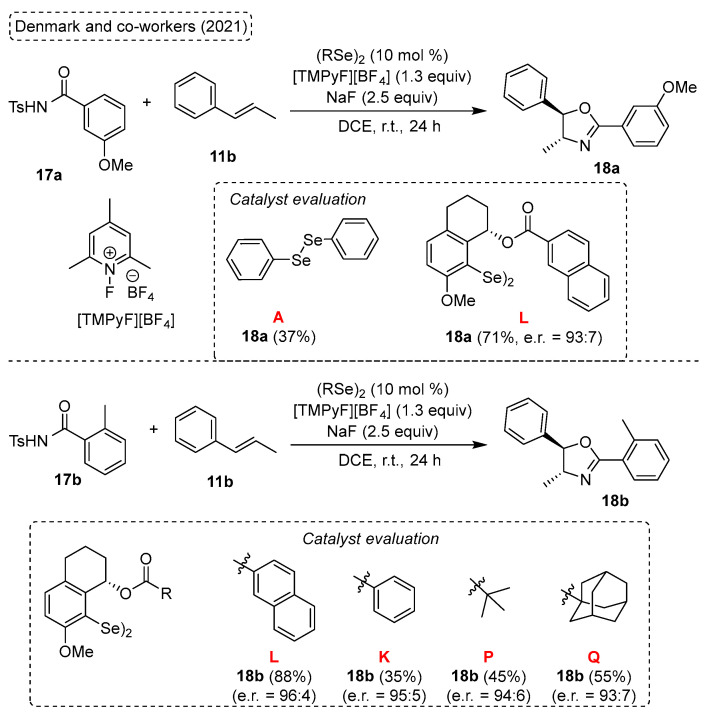
Catalyst evaluation for the synthesis of compounds **18a** and **18b** [31].

The amination of alkenes using *N*-tosyl urea as the nucleophile was previously described by Denmark (Section 2.1) [31]. In 2021, advances using *N*-tosyl amides as nucleophiles were reported by the same research group [34], in which chiral diselenide catalyst **L** was used to promote the chemo-, regio-, diastereo-, and enantioselective 1,2-oxyamination of alkene **11**. Initially, the influence of 10 mol% of diaryl diselenide **A** or **L** was evaluated in the reaction of 3-methoxy-*N*-tosyl benzamide **17a** with (*E*)-prop-1-en-1-ylbenzene **11b** using 1.3 equiv. of [TMPyF][BF_4_] and 2.5 equiv. of NaF in 1,2-dichloroethane (DCE) at r.t. for 24 h. Diphenyl diselenide **A** produced target product **18a** at only 37%, and when the chiral diselenide **L** was used, the desired compound, **18a**, was obtained at a good yield (71%) and excellent e.r. (93:7) (Scheme 13).

Subsequently, the influence of different chiral diselenides (**L**, **K**, **P**, and **Q)** was determined by the reaction of 2-methyl-*N*-tosyl benzamide **17b** with (*E*)-prop-1-en-1-ylbenzene **11b** under the same conditions described above. The change in substituent in the chiral selenium catalyst from 2-naphthoate ester **L** to the benzoate ester **K** derivative did not show a significant decrease in the e.r.; however, the yield of target product **18b** was lower. Similar results were obtained when the selenium catalyst was substituted with alkyl esters, such as *^t^*butanoyl **P** or 2-adamantoyl **Q**. Therefore, the chiral diselenide containing the 2-naphthoate ester **L** substituent was chosen as the best catalyst (Scheme 13).

**Scheme 14:**
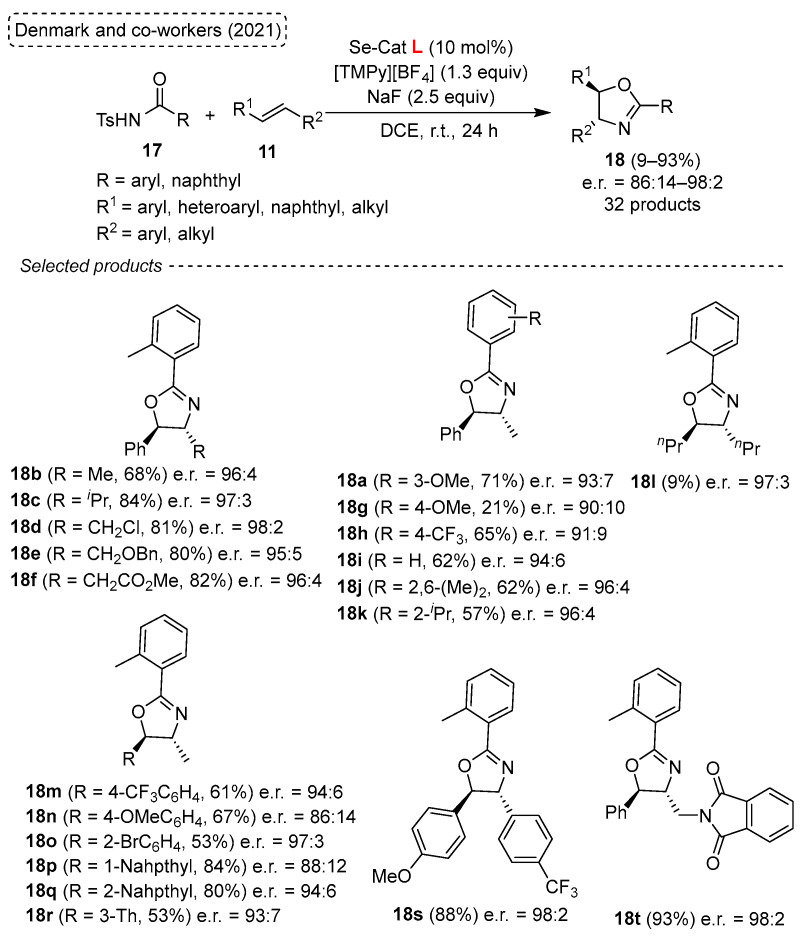
Synthesis of compounds **18** [31].

**Scheme 15:**
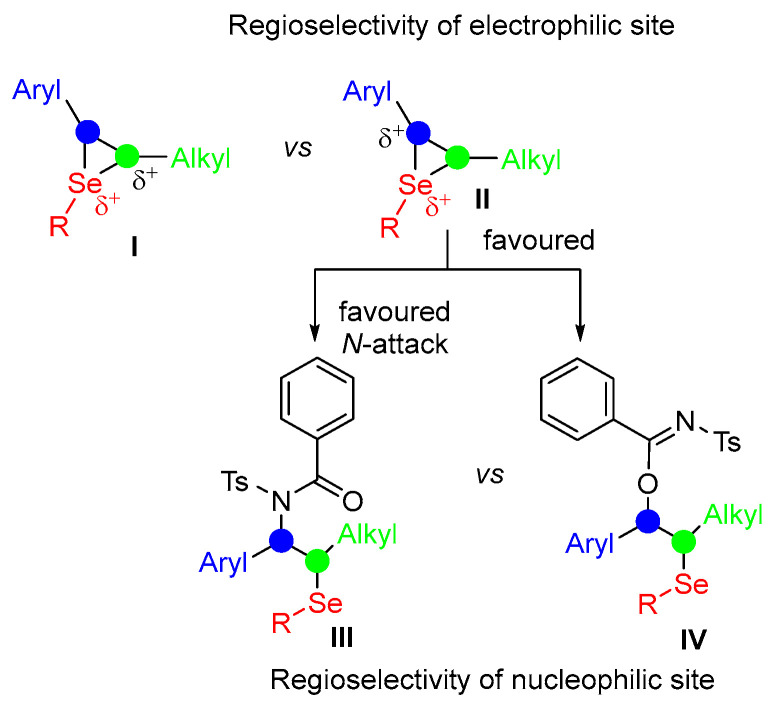
Regioselectivity of the compounds **18**.

The protocol was efficient for a wide range of starting materials: amide **17** substituted by aryl and naphthyl groups, and aryl-alkene **11** substituted by aryl and alkyl (Scheme 14). This variety of starting materials afforded target product **18** (32 compounds) at yields ranging from low to excellent (9–93%). Alkene **11**, containing alkyl-alkyl substituents, gave a poor yield of target compound **18l** (9%); this result is probably due to the low stabilisation of the partial positive charge (δ+) of the selenium intermediate, as shown in Scheme 15. Chiral diselenide **L** showed good efficiency once the synthesised compounds, **18**, were obtained with an e.r. ranging from good to excellent (86:14/98:02), with only one diastereoisomer. Despite the large amount of NaF utilised, the Denmark method presented a wide variety of products with a high e.r. and with high tolerance to different functional groups.

The regio-, diastereo- and enantioselectivity could be explained by substituents in the starting materials, and for this, understanding which carbon is more reactive in the intermediate (seleniranium) is necessary, as well as which amide atom is more nucleophilic (*N* or *O*). The oxyamination could form two possible constitutional isomers, once the seleniranium ion intermediate has two electrophilic sites **I** or **II**, however due to its electronic character the reaction goes preferentially in only one direction. When the alkenes substituted with aryl and alkyl groups are used, the partial positive charge is stabilised by aryl group **II** (more electrophilic site) instead of alkyl one **I**. This possibility can be observed when the unsymmetrical alkene, 1-methoxy-4-(4-(trifluoromethyl)styryl)benzene (starting material of product **18s**), was used, which contained OMe and CF_3_ groups bonded on the aromatic ring. In these cases, the nucleophilic addition occurs in the carbon attached to the aromatic ring containing the methoxy group. The electron donating effect better stabilises the positive charge than 4-trifluoromethyl group. 

Afterwards, the preference of nucleophiles (oxygen **IV** and nitrogen **III**) sites was also proposed, whereby the tosyl group (EWG) bonded to the *N*-tosyl amides, decrease the propensity for delocalization of the nonbonding electron pair from nitrogen for resonance to the carbonyl group (Scheme 15). Still, the 2,4,6-collidine formed in situ can deprotonate the N–H bond due the low *p*K_a_ of this hydrogen. Due these properties, the nitrogen atom of amide is the first nucleophilic **III** in the site capable of supporting the positive charge in the seleniranium ion intermediate **II**.

Both steps, seleniranium ion formation and selenium elimination, are important for the stereoselectivity of this method. Seleniranium ion is formed through a *syn* reaction in a concerted mechanism, which can be further attacked by a nucleophile in an *anti*-fashion reaction. Similarly, the attack of the oxygen atom of the carbonyl group promotes the displacement of Se(IV) intermediate **III**, which goes through an S_N_2-type reaction, attacking the selenium on the opposite site. Based on this proposal, the use of *E* alkene gave the target product in a *trans* relationship while giving *Z* alkene in the *cis* relationship (Scheme 15).

**Scheme 16:**
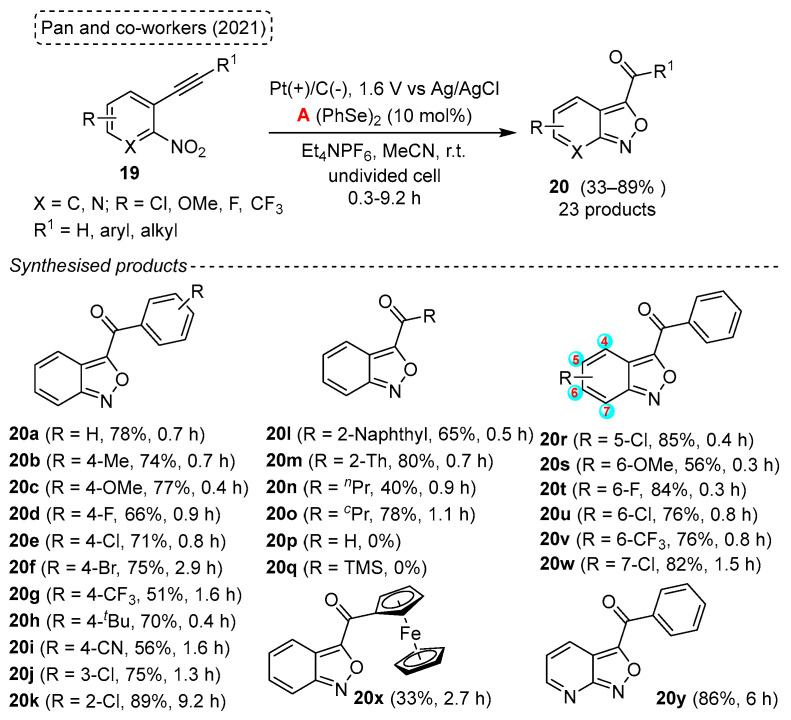
Synthesis of 2,1-benzoxazole **20** [35].

**Scheme 17:**
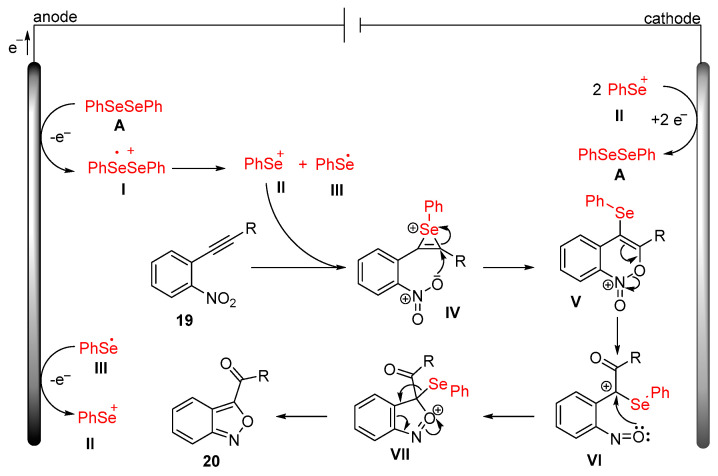
Mechanism proposed for the synthesis of 2,1-benzoxazole **20**.

The use of diorganyl diselenide as a catalyst was efficiently applied to the synthesis of 2,1-benzoxazole **20** from the reaction of *o*-nitrophenylacetylene **19** under electrochemical conditions by Pan and his research group in 2021 (Scheme 16) [35]. In this approach, diphenyl diselenide **A** activates the alkyne to suffer a nucleophilic attack by the nitro group of the starting material, **19**, which is different from traditional methods that reduce the nitro group to nitroso and then carry out the nucleophilic attack (Scheme 17).

In this nucleophilic cyclisation, *o*-nitrophenylacetylene **19** was reacted in the presence of 10 mol% of diphenyl diselenide **A** as the catalyst and 0.5 equiv. of Et_4_NPF_6_ as the electrolyte in CH_3_CN as the solvent at r.t. in an undivided cell containing a graphite rod cathode (Φ 6 mm) and a Pt plate anode (1 cm × 1 cm), with constant potential = 1.6 V vs. Ag/AgCl. When using this system, a wide range of 23 2,1-benzoxazole **20** products were synthesised in yields ranging from moderate to good (33–89%). The protocol was sensitive to the electronic effects of the substituents located on the aromatic ring bonded in the R^1^ position. The presence of EWGs (such as 4-CF_3_ and 4-CN) at this aromatic ring (R^1^) position gave the desired product, **20g** and **20i**, in moderate yields (51% and 56%, respectively) over a longer reaction time (1.6 h) when compared to the starting material substituted with EDGs (such as 4-Me and 4-OMe), which gave the target products, **20b** and **20c**, in better yields and over a shorter reaction time (74%, 0.7 h and 77%, 0.4 h, respectively). The protocol was efficiently extended to the alkyl, 2-naphthyl (**20l**), 2-thienyl (**20m**), and ferrocenyl (**20x**) groups, as well as *o*-nitropyridylacetylene (**20y**) (Scheme 16).

The proposed mechanism for electrochemical cyclisation starts with the reaction of diphenyl diselenide **A** in the anode to form intermediate selenium cation **II** and phenyl selenium radical **III** (which can also form another equivalent of selenium cation **II** by single electron transfer (SET) on the anode). Selenium cation intermediate **II** reacts with alkyne **19** to form the selenonium intermediate **IV**, which is attacked by oxygen on the nitro group in an intramolecular nucleophilic cyclisation (forming the cyclic intermediate **V**). Subsequently, intermediate **VI** is formed after the cleavage of the N−O bond, giving tertiary carbocation **VI**, which is attacked by oxygen to afford intermediate **VII**. Finally, after the release of the selenium cation **II** species, the desired product is formed: **20** (Scheme 17). 

**Scheme 18:**
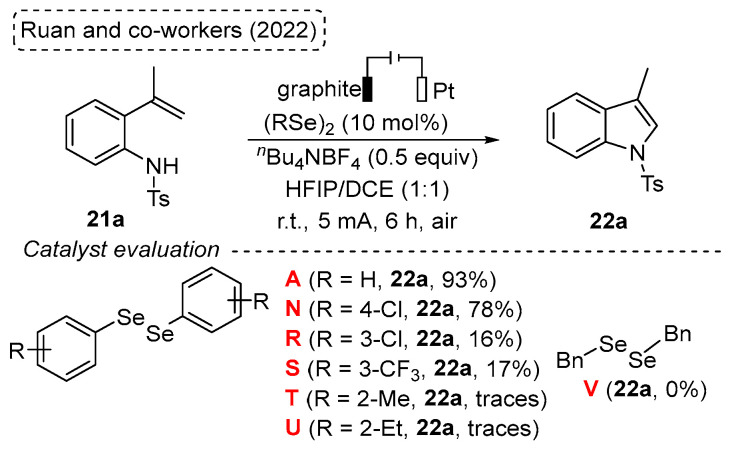
Catalyst evaluation for the synthesis of compounds **22a** [36].

In 2022, Ruan and co-workers [36] applied electrochemistry to perform the intramolecular cyclisation of 2-vinylanilide **21** using diphenyl diselenide **A** as a catalyst to synthesise indole **22**. This selenium catalyst protocol has an advantage over indole synthesis **22** since this sustainable method supports several 2-vinylanilide **21**, containing highly functional, sensitive groups and bioactive compounds (natural molecules, drugs, peptides, and amino acid). Firstly, diaryl diselenides **A**, **N**, and **R**–**V** were screened using 2-vinylanilide **21a** as starting material. When dibenzyl **V** or *ortho-*substituted (methyl **T** or ethyl **U**) diaryl diselenides were evaluated, only traces of target indole **22a** were obtained. When the *meta*-substituted (Cl **R** or CF_3_ **S**) diaryl diselenides were tested, target compound **22a** was obtained at a poor yield (16% and 17%, respectively). The best yields were obtained when 1,2-bis(4-chlorophenyl) diselenide **N** and diphenyl diselenide **A** were used; in these cases, the desired indole, **22a**, was formed at good yields (78% and 93%, respectively). For this intramolecular C*_sp_*_2_–H amination, the best conditions were established when the 2-vinylanilide **21** was reacted in the presence of 10 mol% of diphenyl diselenide **A** as the catalyst, *^n^*Bu_4_NBF_4_ (0.1 M), 1,1,1,3,3,3-hexafluoro-2-propanol (HFIP)/DCE (1:1), and a constant current = 5 mA for 6 h at 23 °C under air conditions in an undivided cell system containing a graphite anode (1.0 cm × 1.0 cm × 0.2 cm) and a Pt cathode (1.0 cm × 1.0 cm × 0.01 cm) (Scheme 18). 

**Scheme 19:**
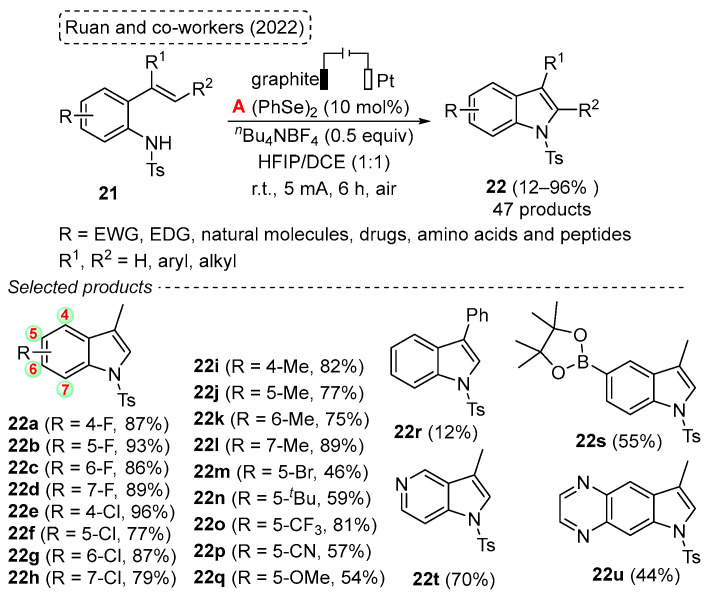
Synthesis of indole **22** [36].

Under this organic redox catalysis method, 47 highly functionalised indoles **22** were obtained at yields ranging from moderate to excellent (12–96%). The protocol was not sensitive to several substituents, such as chlorine, bromo, cyan, pyridyl, pinacol ester, and carboxyl groups, and also tolerated starting materials, **21**, containing active hydrogens such as sulfonamide, free carboxylic acid, and amide, as well as heterocyclic moieties (thiazole or pyrazine). An unsatisfactory result was obtained when the methyl group (R^1^) bonded to the vinyl substrate **21** was changed to phenyl, and in this case, 3-phenyl indole **22r** was obtained at only a 12% yield (Scheme 19).

**Scheme 20:**
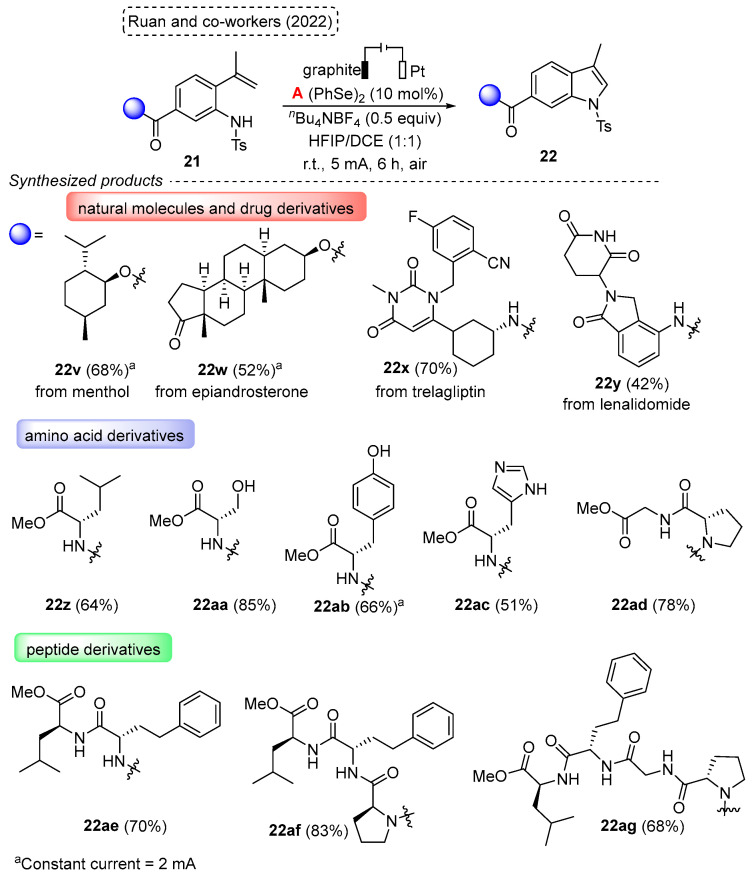
Synthesis of indole **22** containing natural molecules and drug derivatives [36].

The indoles containing menthol **22v** or epiandrosterone **22w** natural substituents were obtained at moderate yields (68% and 52%, respectively). Similar results were obtained when the starting materials containing drug derivatives were reacted under standard conditions; in these cases, the indoles containing trelagliptin (a drug for type 2 diabetes) portion **22x** and lenalidomide (a drug for multiple myeloma) moieties **22y** were obtained at 70% and 60%, respectively. Still, the method was applied to amino acids and peptides, obtaining target products **22z**–**ad** without racemisation. The method was efficient with several starting materials containing serine, tyrosine, and histidine derivatives, as well as dipeptide, tripeptide, and tetrapeptide, which afforded the highly functionalised indole **22z**–**ag** (8 products) at yields ranging from moderate to good (51–85%) (Scheme 20).

**Scheme 21:**
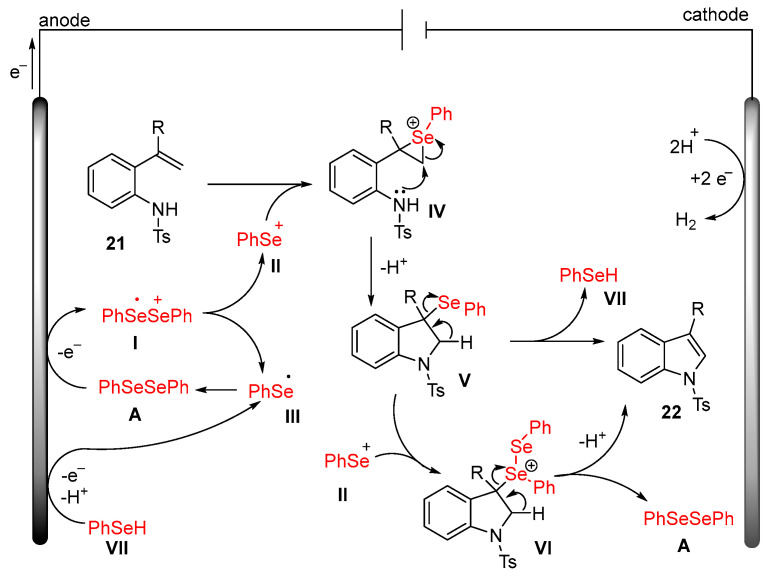
Mechanism proposed for the synthesis of indole **22**.

The mechanism proposed by the authors starts with the formation of selenium cation **II** and radical **III** intermediates via a reaction of diphenyl diselenide **A** with the anode. Afterwards, cation intermediate **II** reacts with the alkene **21** portion to form seleniranium cation **IV**, which can be attacked by nitrogen atoms to obtain cyclic intermediate **V**. This intermediate **V** has two routes to form the indole of interest, **22**; the first one occurs after deprotonation and the selenium (selenophenol **VII**) elimination step; in the second route, intermediate **V** reacts with another selenium cation **II** to give intermediate **VI**, which produces the desired indole, **22**, and regenerates selenium catalyst **A** to a new cycle after the elimination step. In this protocol, it was not necessary to use sacrificial chemical oxidants, since the protons previously eliminated in the aromatisation step undergo cathodic reduction to generate H_2_. Still, the selenophenol **VII** formed can be oxidised to selenium radical **III** in the anode, and selenium radical **III** is easily dimerised to induce the catalyst diphenyl diselenide **A** to begin a new catalytic cycle (Scheme 21). 

**Scheme 22:**
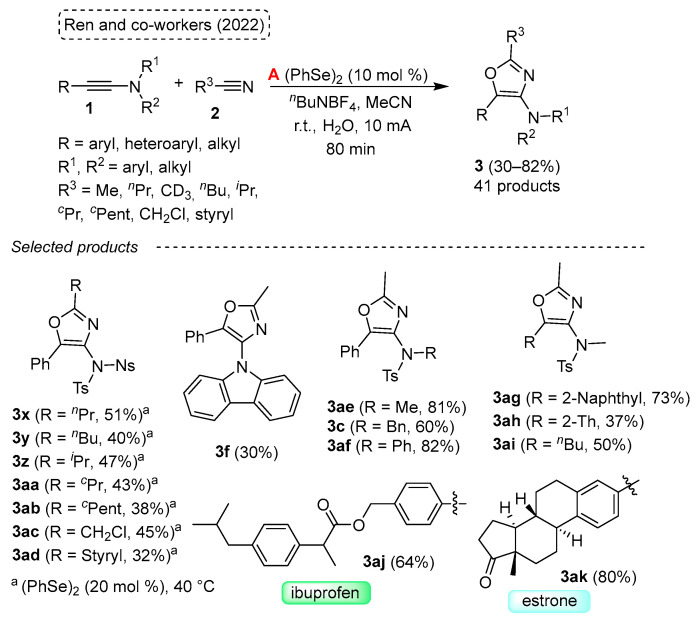
Synthesis of oxazole **3** [37].

In 2022, Ren and co-workers described [37] the use of electrochemistry as a sustainable alternative for the synthesis of polysubstituted oxazole **3** by the *N*,*O*-difunctionalisation of ynamide **1** using diphenyl diselenide **A** as a catalyst. A green, efficient, and high atom economy method was reported, in which several ynamides, **1**, were reacted in the presence of 10 mol% of diphenyl diselenide **A** as a catalyst, 1.5 equiv. of *^n^*Bu_4_NBF_4_, and organyl nitrile **2** as the solvent in nitrogen at r.t. for 80 min in an undivided cell composed of a carbon graphite cloth (10 mm × 10 mm) cathode and a carbon graphite cloth (10 mm × 10 mm) anode at a constant current of 10 mA. The principal advantage of the optimised method is that it does not require an external chemical oxidant (Scheme 22).

Under these conditions, several *N*-protected as well as aryl/alkyl alkynyl-substituted ynamides, **1**, were reacted, giving a wide range of target oxazoles, **3** (41 compounds), at yields ranging from low to good (30–82%). Oxazole **3** substituted with ibuprofen and estrone derivatives **3aj** and **3ak** was synthesised using standard conditions with yields of 64% and 80%, respectively. The protocol was extended to nitrile **2** substituted with alkyl (Me, *^n^*Pr, CD_3_, *^n^*Bu, *^i^*Pr, *^c^*Pr, *^c^*Pent, and CH_2_Cl) and styryl groups (compounds **3x**–**ad**); however, in these cases, it was necessary to increase diphenyl diselenide catalyst **A** from 10 mol% to 20 mol%, and the reaction was performed at 40 °C instead of r.t. (Scheme 22).

**Scheme 23:**
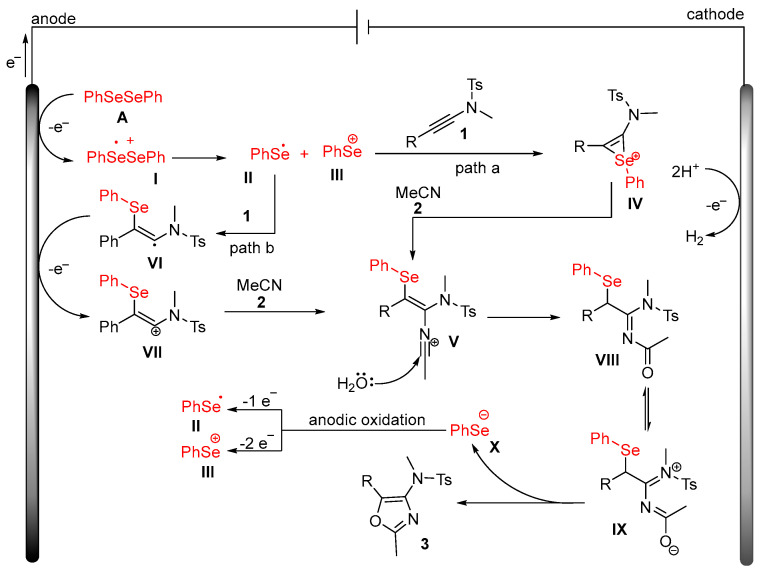
Mechanism proposed for the synthesis of oxazole **3**.

The mechanism for the synthesis of target oxazole **3** proposed by the authors starts with the cleavage of the Se–Se bond, forming the phenyl selenium radical **II** and cation **III** species. After that, there are two possible pathways. The first is anionic, in which the cation **III** intermediate is reacted with starting material **1** to obtain intermediate **IV**. Intermediate **VII** can be formed by the radical species of selenium compound **II** and starting material **1**. Subsequently, intermediates **IV** or **VII** suffer a nucleophilic attack from acetonitrile **2** to give intermediate **V**, which can be hydrolysed to form intermediate **VIII**. Intermediate **VIII** is in equilibrium with the zwitterionic tautomer intermediate **IX**. Finally, the target oxazole **3** is formed by C–Se bond cleavage and deprotonation steps, forming the phenyl selenium anion **X**. Intermediate **X** undergoes anodic oxidation to regenerate electrophilic phenyl selenium species **II** and **III** for a new cycle (Scheme 23).

**Scheme 24:**
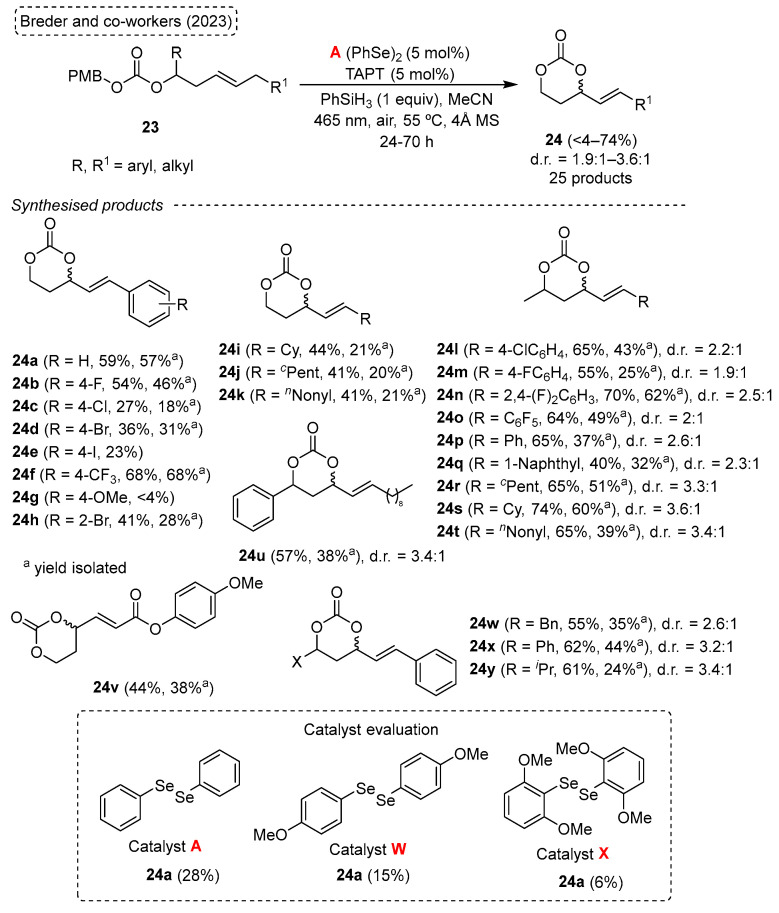
Synthesis and catalyst evaluation for the obtention of cyclic carbonate **24** [38].

More recently, in 2023, Breder and his research group [38] described the advances in the use of photo-aerobic selenium-*π*-acid multicatalysis in the synthesis of cyclic carbonate **24**. The method was developed through the reaction of non-activated homoallylic carbonate ester **23** under ambient air as the terminal oxidant, using visible light as an energy source (Scheme 24). In this approach, pyrylium dye and diphenyl diselenide **A** were used for the activation of the C=C double bond of the homoallylic carbonic acid esters, allowing for the intramolecular attack of nonprotic nucleophiles. In this regard, the authors reported that the reaction using carbonate substituted with *p*-methoxybenzyl (PMB) was better when compared to the *^t^*Bu group for the increase in nucleophilicity of the oxygen atom of carbonate. Moreover, the selenium catalysts were tested, such as diphenyl diselenide **A**, (4-MeOC_6_H_4_Se)_2_ **W**, and (2,6-(MeO)_2_C_6_H_3_Se)_2_ **X**, and the best result was obtained when diphenyl diselenide **A** was used (Scheme 24).

Under this approach, a wide range of 4-mono- and 4,6-disubstituted 1,3-dioxan-2-one **24** (25 products) were synthesised at yields ranging from low to good (<4–74%) with a diastereomeric ratio (d.r.) of 3.6:1–1.9:1. These target compounds were obtained via the intramolecular reaction of carbonate **23** in the presence of 5 mol% of diphenyl diselenide **A** as a catalyst, 5 mol% of TAPT, 1 equiv. of PhSiH_3_, and MeCN as the solvent with 465 nm irradiation under an air atmosphere at 55 °C using 4Å molecular sieves (MS) and a reaction time of 24 h to 70 h. The new protocol, as described by Breder, tolerated several starting materials, **23**, substituted with alkyl and aryl, and when 4,6-disubstituted dioxanone **24** was obtained, the *cis*-diastereoselectivity was preferable. In all cases, a mixture of enantiomers was obtained; however, the authors did not use catalysts containing a chiral moiety, which could induce an enantiomer excess (Scheme 24).

**Scheme 25:**
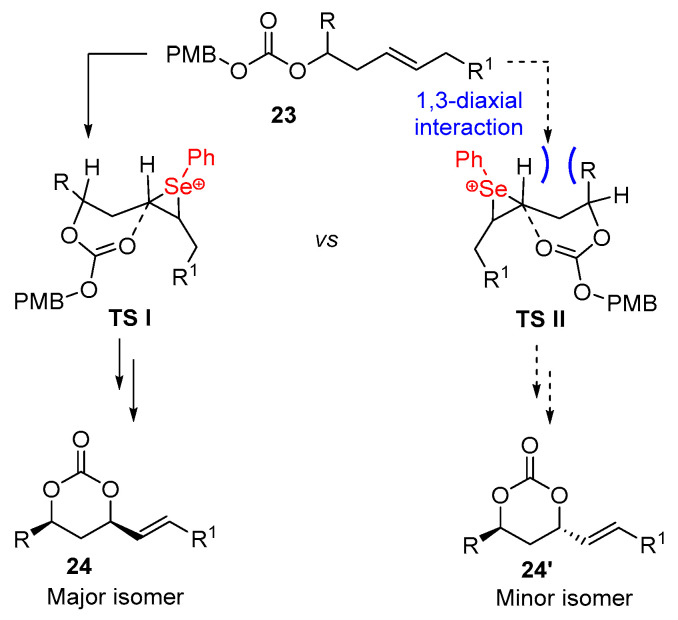
Selectivity in the synthesis of cyclic carbonate **24**.

The authors propose stereoselectivity for the reaction based on the transition state (TS) geometries, **TS I** and **TS II**, as demonstrated in Scheme 25. For **TS II**, where the carbonate terminal substituent is preferentially in a pseudo-equatorial orientation, there is a greater 1,3-diaxial interaction (Scheme 25). This 1,3-diaxial interaction did not occur for **TS I**. This selectivity increases with the increasing steric hindrance of the R group, as observed according to the d.r. of the synthesised **24p** (R = Me, d.r. = 2.6:1) and **24w** (R = Bn, d.r. = 2.6:1) compounds, which were formed under a minor d.r. since they are less sterically hindered than the **24x** (R = Ph, d.r. = 3.2:1) and **24y** (R = *^i^*Pr, d.r. = 3.4:1) (Scheme 24).

**Scheme 26:**
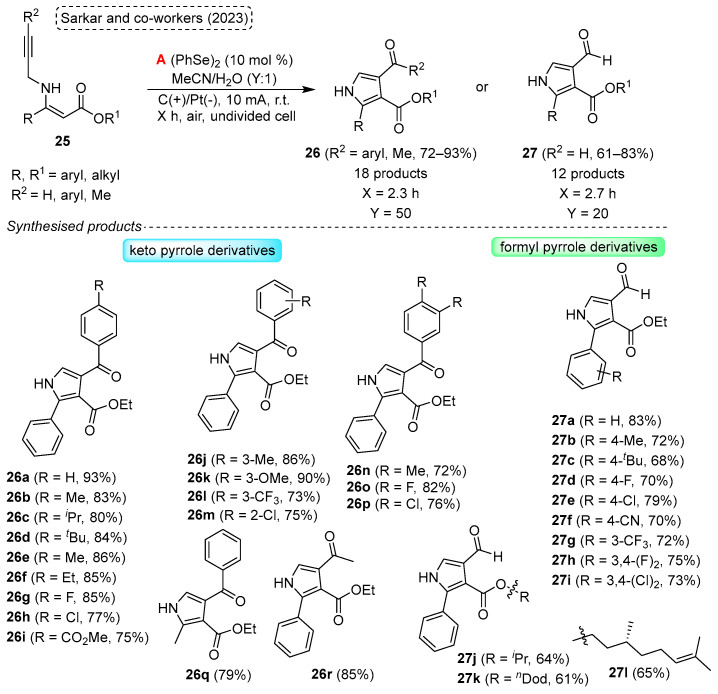
Synthesis of pyrroles **26** and **27** [39].

Recently (2023), Sarkar and co-workers [39] described the synthesis of carbonyl-pyrroles **26** or **27** or -oxazole **5** using diphenyl diselenide **A** as an electrocatalyst, which activates the alkyne portion of the *N*-propargyl derivatives (enamines **25** or amides **4**). This is a sustainable redox process that promotes an intramolecular electro-oxidative addition reaction activated by a cationic phenyl selenium species (Scheme 26). 

In this method, *N*-propargyl enamine carboxylate **25** (alkyne substituted with aryl or Me groups) was reacted with 10 mol% of diphenyl diselenide **A** as a catalyst in a solvent mixture of MeCN/H_2_O (50:1) and LiClO_4_ (0.1 M) as the electrolyte. Moreover, electrochemistry apparatus containing a graphite plate as the anode and a platinum sheet as the cathode in an undivided cell was used, with a constant current of 10 mA at r.t. under an air atmosphere for 2.3 h. Under these conditions, the target 4-carbonylpyrroles (keto-pyrroles) **26** were obtained (18 compounds) at yields ranging from good to excellent (72–93%) (Scheme 26). In this study, the authors did not use other diselenides to perform a reactivity comparison.

The protocol was not sensitive to starting material **25** containing EWGs or EDGs attached at different positions of the aryl-substituted portion in the alkyne, as well as being attached to a disubstituted aromatic ring. The less reactive alkyl-substituted alkyne or alkyl alkenyl enamine were efficiently reacted under the above conditions, giving the desired products **26r** and **26q** at 85% and 79% yields, respectively (Scheme 26). 

When the terminal alkynes on *N*-propargyl enamine carboxylate **25** were used as the starting material to synthesise 4-formylpyrroles **27**, the previous conditions were adapted, requiring a decrease in the amount of mixture of MeCN/H_2_O from (50:1) to (20:1), as well as an increase in the time of the reaction from 2.3 h to 2.7 h. Under these conditions, target 4-formylpyrrole **27** (12 products) was obtained at good yields (61–83%). Moreover, the protocol was used to obtain the pyrroles **27l** and **27k**, which were substituted with naturally occurring derivatives, such as citronellol and long-chain alcohols (Scheme 26).

**Scheme 27:**
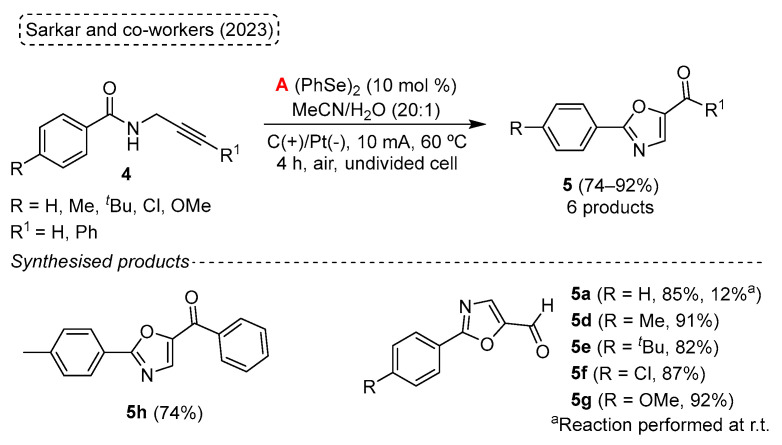
Synthesis of oxazole **5** [39].

The authors extended the electrochemistry method by using diselenide as the catalyst for *N*-propargyl amide **4** to synthesise 5-carbonyl oxazole **5**, but higher temperature and longer reaction times were necessary: 60 °C and 4 h. Under these conditions, 5-carbonyl oxazole **5** was obtained (6 compounds) at yields ranging from good to excellent (74–92%). In general, 5-formyl oxazole **5** was obtained with higher yields when compared to 5-benzoyl oxazole, e.g., the **5d** and **5h** formyl and benzoyl derivative compounds were isolated at 91% and 74%, respectively (Scheme 27).

**Scheme 28:**
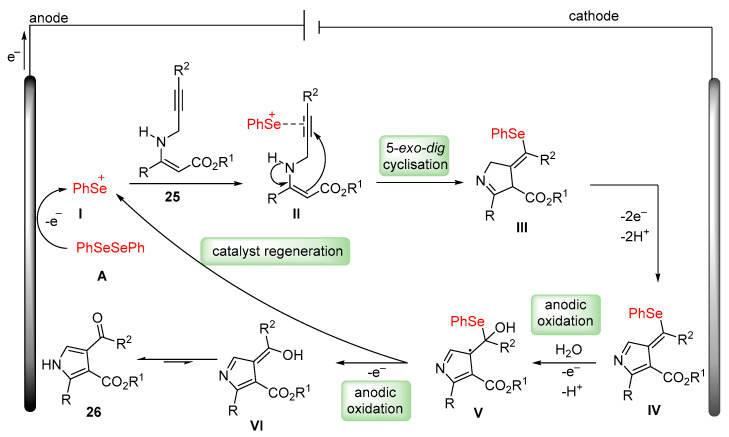
Mechanism proposed for the synthesis of pyrrole **26**.

The plausible mechanism for the synthesis of 4-carbonylpyrrole **26** by selenium-catalysed oxidative cyclisation starts with the formation of the *π*-acidic phenyl selenium cation **I** (PhSe^+^) intermediate from the reaction of diphenyl diselenide **A** with the anode. Afterwards, intermediate **I** interacts with the starting material, *N*-propargyl enamine carboxylate **25**, forming intermediate **II**, which, after the intramolecular nucleophilic addition of enamine in the active alkyne (5-*exo-dig* cyclisation), gives intermediate **III** (exocyclic vinyl selenoether). Intermediate **III** reacts with the anode to obtain the more stable seleno-intermediate **IV**. In the next step, intermediate **IV** reacts with the anode (oxidation), followed by the addition of water, leading to the formation of radical intermediate **V**, after a SET, afforded enol **VI** as well as the regeneration of PhSe^+^ **I** (active catalyst) for a new reactional cycle. The formed Enol **VI** has its balance shifted to the formation of target product **26**. The formation of oxazole **5** is similar to the mechanism described for 4-carbonylpyrrole **26** (Scheme 28).

### 2.2. Addition and Oxidative Functionalisation Reactions

In this section, we describe the reactions involving the additions and oxidations for alkynes, alkenes, or heterocycles. Such reactions with diorganyl diselenide usually need an extra oxidising agent, most commonly Selectfluor^®^, pyridinium triflate salts, or TEMPO. The effectiveness of a catalyst is shown by its ability to react with a broad scope of oxidising agents and with different substrates. In general, similar properties of Se catalysts can be observed in the methodologies selected for this section; however, diphenyl diselenide was the most utilised in the protocols.

**Scheme 29:**
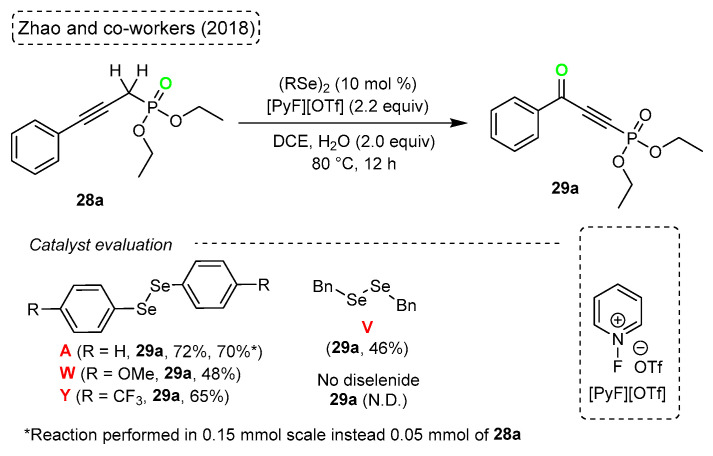
Catalyst evaluation for the synthesis of ynone **29a** [28].

In 2018, Zhao and co-workers [28] described the synthesis of ynones **29** and **31** through an unusual C≡C triple bond migration using propargylphosphonate **28** and 3-alkynoate **30**, with the diaryl diselenide species used as catalysts to promote the conversion of C*_sp_*_3_−P to C*_sp_*−P bonds. Initially, some conditions were established, and the diorganyl diselenide (**A**, **V**, **W**, **Y**) species were evaluated by the authors, for which diethyl (3-phenyl-2-propynyl)phosphonate **28a** was reacted (as the starting material) in the presence of 2.2 equiv. of 1-fluoropyridinium triflate [PyF][OTf], DCE as the solvent, and a 2.0 equiv. of H_2_O at 80 °C for 12 h in the presence of 10 mol% of different diselenides (**A**, **V**, **W**, **Y**). When bis(dibenzyl) or bis(*p*-anisyl) diselenide **V** and **W** were tested, the target product, **29a**, was obtained at moderate yields, 46% and 48%, respectively (Scheme 29). Despite the low yield, it is necessary to highlight that bis(dibenzyl) diselenide was able to promote the reaction, unlike the other methods (Scheme 29). 

The presence of neutral or EWGs at the aromatic ring of diaryl diselenide **A** and **Y** improved the yield of **29a**. When 1,2-bis [4-(trifluoromethyl)phenyl]diselenide **Y** was used, the **29a** compound was obtained at 65%, and the best catalyst for this reaction was diphenyl diselenide **A**, which formed target product **29a** at 72%. Furthermore, the authors performed a test in the absence of a catalyst, and compound **29a** was not detected, showing its importance in the reaction.

**Scheme 30:**
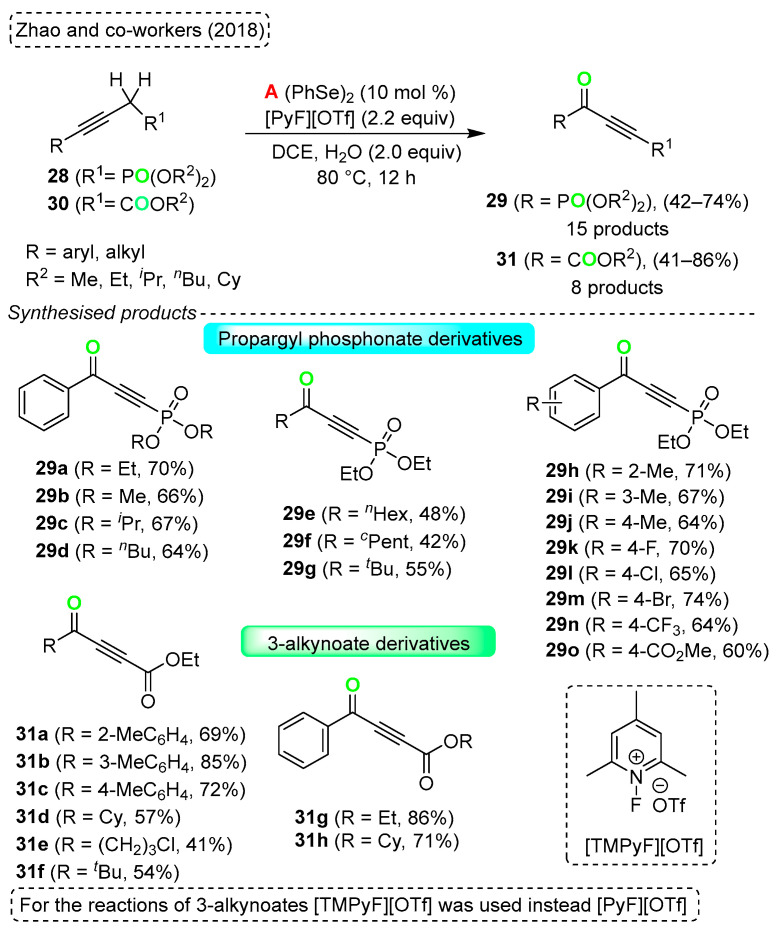
Synthesis of ynone **29** [28].

In this approach, several 3-substituted propargyl phosphonates, **28**, with alkyl and aryl groups containing EWGs and EDGs in different positions of the aromatic ring, as well as alkyl substituents (Me, Et, *^i^*Pr, *^n^*Bu) in the phosphonate **28** portion, were used as the starting materials, which gave target ynone **29** (15 products) at yields ranging from moderate to good (42–74%). This protocol was efficiently extended to 3-alkynoate derivative **30**, which afforded the desired ynone **31** (8 compounds) at slightly higher yields (41–86%) than propargyl phosphonate derivative **29**. The method was not sensitive to both the electronic and steric effects of the substituents of starting material **28** (Scheme 30).

**Scheme 31:**
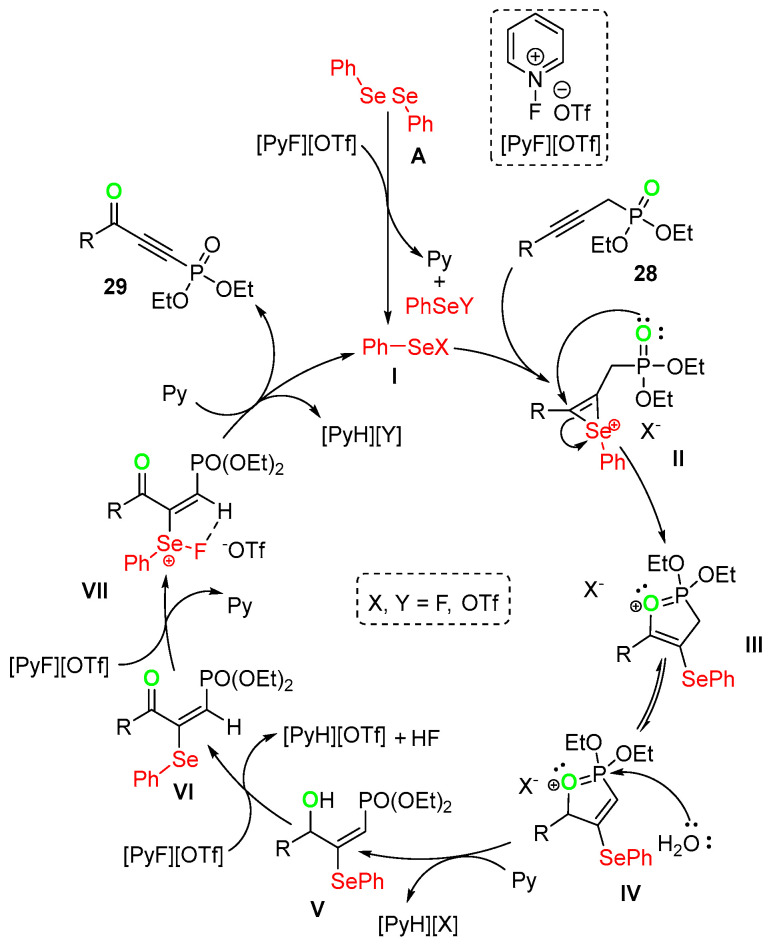
Mechanism proposed for the synthesis of ynone **29**.

The mechanism proposed by the authors starts with the reaction of diphenyl diselenide **A** with [PyF][OTf] to promote the formation of electrophilic species PhSeX **I**, and subsequently, the reaction of alkyne **28** with intermediate **I** forms seleniranium ion **II**, which is attacked by the phosphonate group to give cyclic intermediate **III**. Intermediate **III** is in equilibrium with **IV**, which is attacked by H_2_O (in the presence of base) to form alcohol intermediate **V**. Next, alcohol **V** is oxidised to produce ketone intermediate **VI**, and cationic intermediate **VII** suffers an oxidative *syn* elimination of the selenium portion and hydrogen (through deselenenylation via C*_sp2_*−Se bond cleavage) to form the desired ynone **29**, and catalyst **I** is regenerated for a new cycle (Scheme 31).

**Scheme 32:**
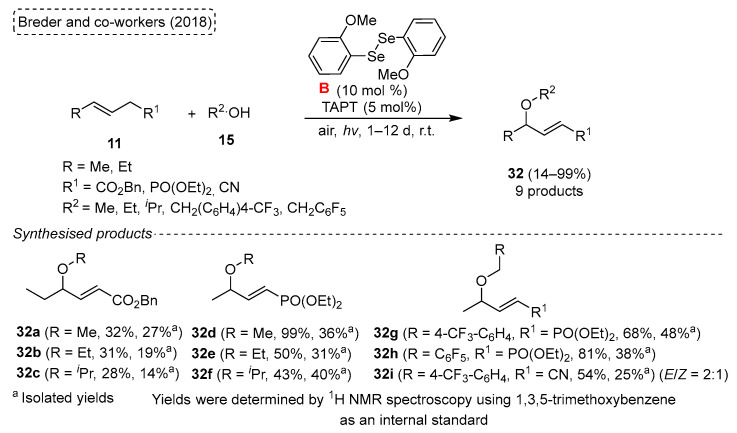
Synthesis of noncyclic ether **32** [29].

The previously optimised conditions, as described by Breder in 2018, were adapted for the formation of noncyclic ether **32** via the intermolecular reactions of alcohol **15** with alkene **11** [29]. In this case, an increase in the loading of Se-catalyst **B** from 5 mol% to 10 mol% and TAPT from 3 mol% to 5 mol% were necessary. Under these conditions, the desired ether **32** (9 products) was generally obtained at moderate yields after a very long reaction time (many days, 1–12 d). It is possible that these results are due to the long time of the reactions, which may have caused a significant loss of olefinic starting material **11** (Scheme 32).

**Scheme 33:**
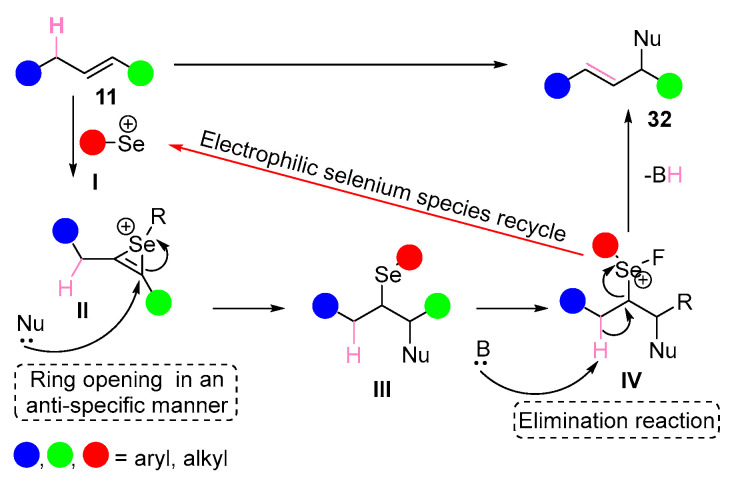
Proposed mechanism for the oxidative functionalisation of alkenes.

The mechanism for the oxidative functionalisation of alkenes occurs in a similar way. Initially, selenium electrophilic species **I** reacts with alkene **11** to form seleniranium intermediate **II**. Intermediate **II** suffers from a nucleophilic attack to give compound **III** by a ring opening in an *anti*-specific manner. Afterwards, the *anti β*-elimination of **IV** occurs under oxidative conditions, recycling electrophilic selenium species **I** for a new cycle, and the target **32** derivative compound is formed from allylic functionalisation (Scheme 33).

In 2018, Zhao and co-workers [40] developed an appropriate approach for oxidative allylic fluorination catalysed by organoselenium compounds. The oxidant and fluorine source in this process was the bulky electrophilic fluorinating reagent *N*-fluoro-2,4,6-trimethylpyridinium triflate [TMFP][OTf]. Interestingly, TEMPO, as an additive, increases the fluorination rating, resulting in an improved substrate scope and functional group tolerance.

**Scheme 34:**
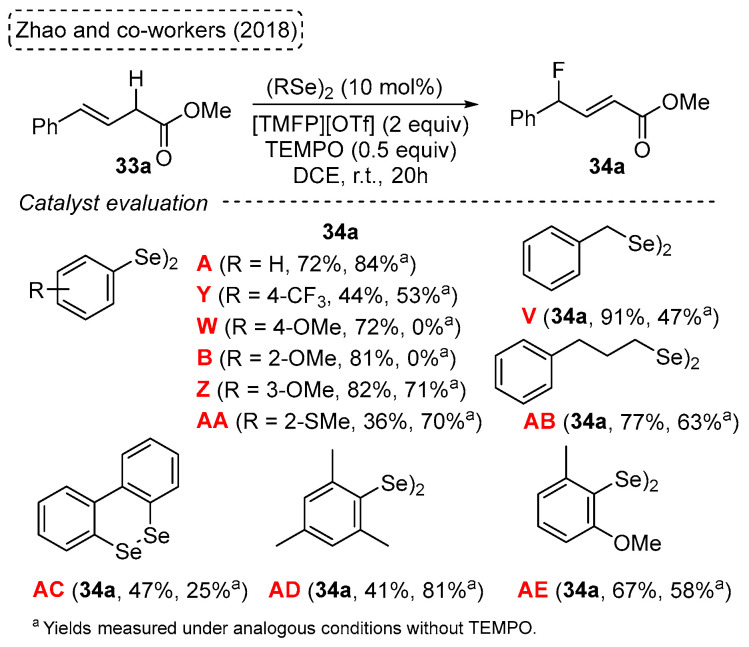
Catalyst evaluation for the synthesis of compound **34a** [40].

Several diselenides were explored in an effort to ramp up the oxidation of the selenide moiety in the intermediates, focused on improving the efficiency of the turnover-limiting deselenenylation process. Additionally, to avoid catalyst degradation, different additives were examined further, and it was shown that allylic fluoride **34a** could be produced in the highest yield when BnSeSeBn (**V**) was reacted with TEMPO (0.5 equiv.) and [TMFP][OTf] (2 equiv.). This is the first study in which dibenzyl diselenide worked efficiently, and its effect on other starting materials was investigated. In general, the dialkyl diselenides were more efficient than the diaryl diselenides (Scheme 34).

**Scheme 35:**
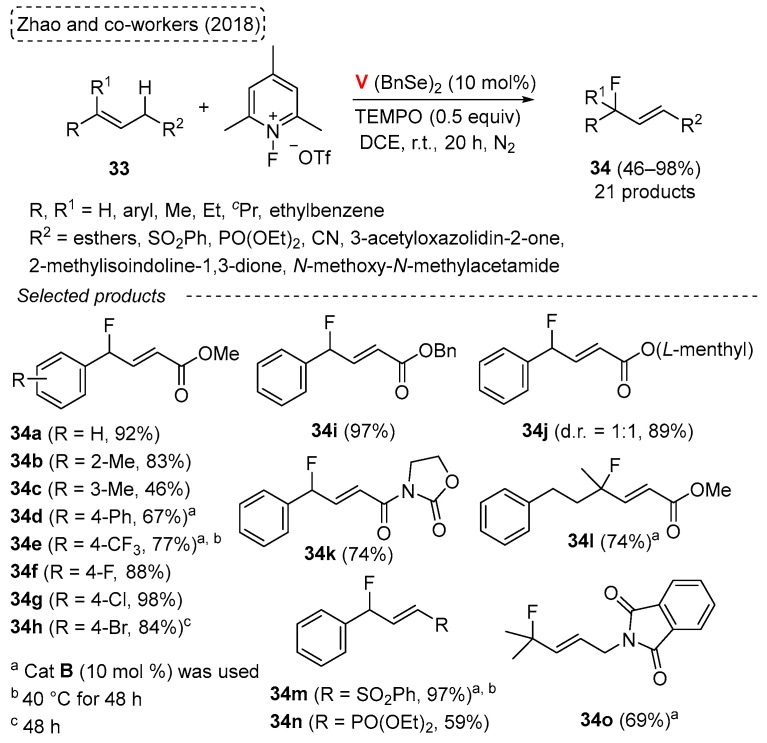
Synthesis of compound **34** by oxidative allylic fluorination [40].

By using the developed method, the authors were able to produce 21 products of allylic fluoride **34**, with yields ranging from 46% to 98%. The reaction was efficient for the various functional groups in substrate **33**, such as olefinic esters, amides, sulfones, phosphonates, and nitriles (Scheme 35). In order to achieve greater yields, electron-rich diselenide catalyst **B** was utilised in the reaction for the **34d**, **34e**, **34l**, **34m**, and **34o** compounds.

**Scheme 36:**
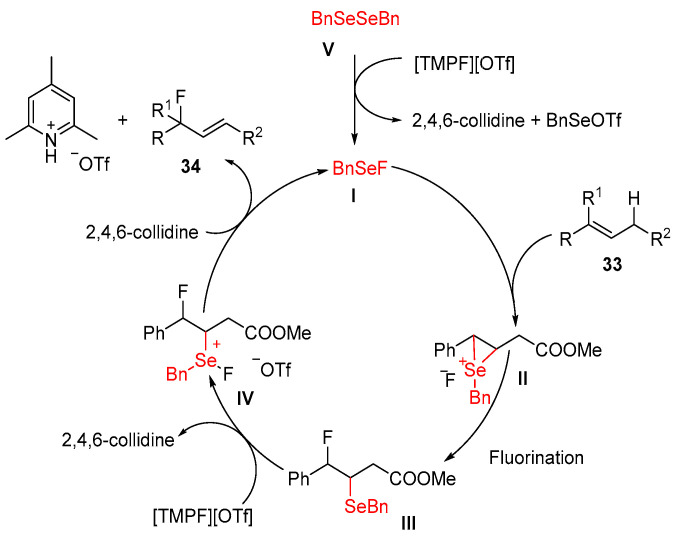
Mechanism proposed for the synthesis of compound **34**.

The mechanism proposed by the authors begins with the oxidative cleavage of diselenide **V**, which releases the electrophilic species BnSeF **I** and BnSeOTf. Seleniranium ion **II** is formed when BnSeF **I** interacts with olefinic ester **33**. Fluoroselenenylated intermediate **III** is generated after ring-opening, which is assisted by the nucleophilic attack of fluorine anions. The BnSe group on intermediate **III** is then oxidised by [TMFP][OTf], resulting in the production of intermediate **IV**. Next, a further H-elimination with the aid of a weak base generates the desirable product, **34**, and regenerates the reactive species, BnSeF **I**, for a new cycle (Scheme 36).

**Scheme 37:**
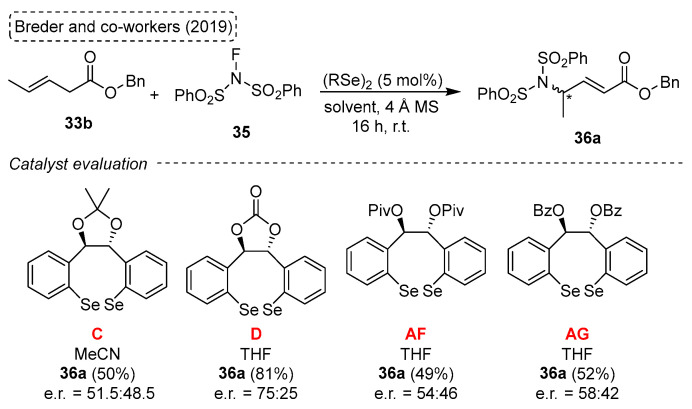
Catalyst evaluation for the synthesis of compound **36a** [30].

Breder and co-workers [30], in 2019, described the synthesis and application of several chiral selenium-*π*-acid catalysts in the asymmetric oxidative functionalisation of alkenes. The synthesised Se-catalysts were applied in intermolecular enantioselective imidation under thermic conditions, as well as for enantioselective aerobic lactonisation under photoredox conditions. In the Breder protocol, selenides and diselenides were tested; however, in this review, only the diselenides are reported (Scheme 37).

Initially, the enantioselective imidation of alkene was evaluated by the authors, and equivalent amounts of benzyl pent-3-enoate **33b** and *N*-fluorobenzenesulfonimide **35** were reacted in the presence of 5 mol% of different diselenides as the catalysts (4 Å MS at r.t. for 16 h). The cyclic diselenides, **C**, **D**, **AF**, **AG**, containing protected 1,2-diols were evaluated; when the 1,2-diols were protected, with acetal **C** (derivative of acetone) used as the catalyst, product **36a** was obtained at a moderate yield (50%) and enantiomeric ratio (51.5:48.5). Similar results were observed when diselenides containing the pivaloyl **AF** and benzoyl **AG** protective groups were tested, and in these cases, product **36a** was formed at yields of 49% and 52%, as well as 54:46 and 58:42 for e.r., respectively. On the other hand, when cyclic diselenide **D** (containing the protected 1,2-diols with a carbonate group) was checked, and an increase in both the yield (81%) and e.r. 75:25 was observed for target product **36a**. This study was limited because the reaction scope of the method was not investigated for other starting materials (Scheme 37).

**Scheme 38:**
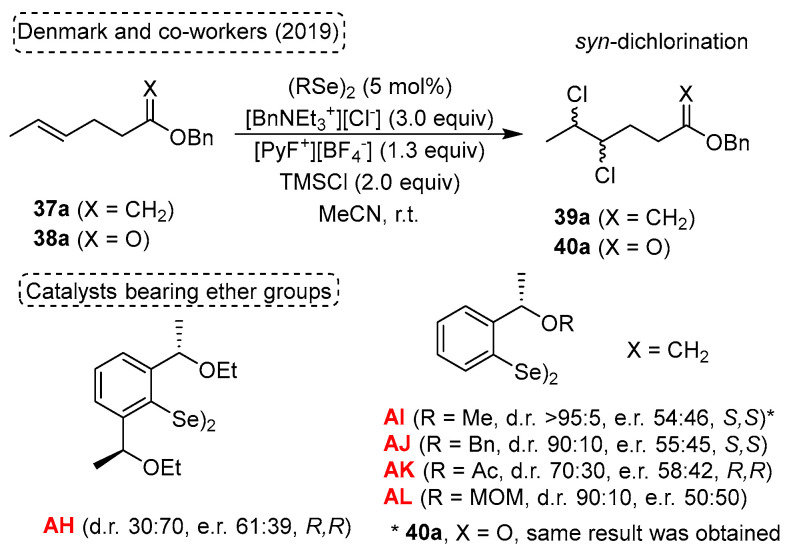
Catalyst evaluation for the synthesis of compound **40a** by *syn*-dichlorination of alkenes [41].

In 2019, Denmark and co-workers [41] developed a study based on an enantioselective *syn*-dichlorination of alkenes **37** or **38**. In this work, 23 diselenides were evaluated as catalysts for the reaction. Firstly, the authors started studying different diselenides containing ether groups, **AH**–**AL**, in the reaction to obtain compound **39a**. When utilising diselenide **AH**, the chlorination formed *syn*-dichloride under an e.r. of 61:39, showing that chiral diselenides can catalyse enantioselective reactions. The other diselenides that were evaluated showed better reactivity but lower enantioselectivity, reaching a maximum e.r. of 58:42 (**AK**) (Scheme 38).

**Scheme 39:**
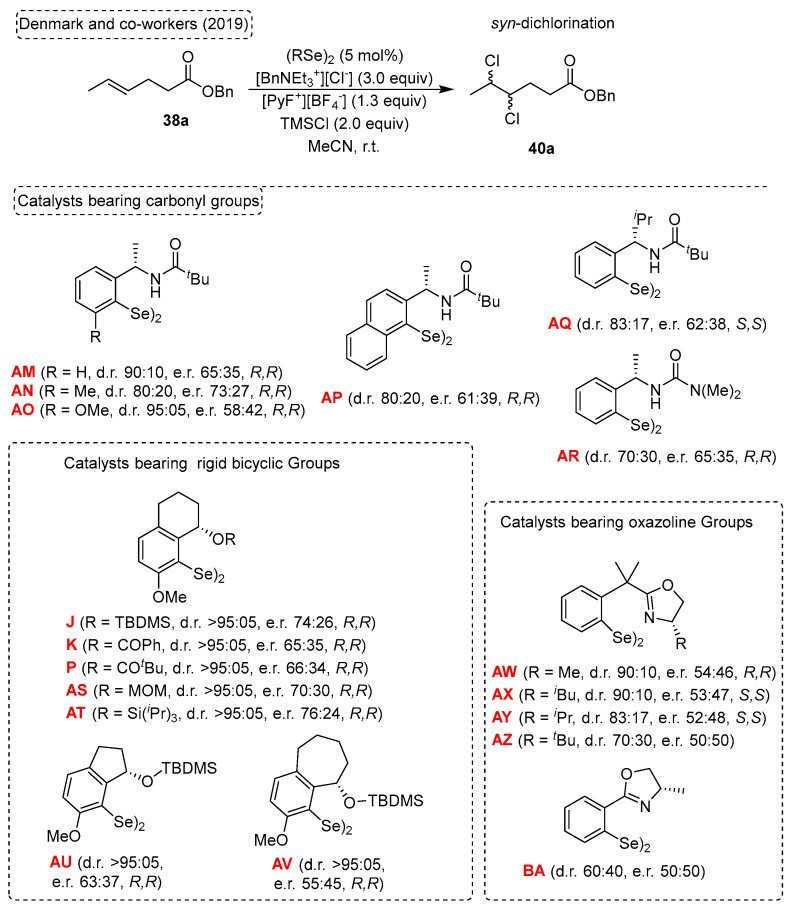
Catalyst evaluation for the synthesis of compound **40a** via the *syn*-dichlorination of alkenes [41].

The carbonyl oxygen in the diselenide containing urea **AR** acts as an alternative co-ordinating group, generating what is anticipated to be a seven-membered ring. Enantiomeric ratios that were greater than the previously recorded (catalyst bearing ether groups) values were found in the dichloride products made with diselenides **AM**–**AR** (catalyst bearing carbonyl groups), with *syn*-addition at a ratio of up to 73:27. In addition, the enantioselectivity improved when diselenide **AN** was employed, but there was a corresponding reduction in diastereoselectivity (Scheme 39). The diselenides bearing oxazoline groups, **AW**–**BA**, showed poor enantioselectivity, producing ratios near that of the racemic mixture but with good diastereoselectivity, with ratios reaching 90:10 (Scheme 39). Moreover, diselenides containing bicyclic groups, **J**, **K**, **P**, **AS**–**AV**, were evaluated, showing the best results for *syn*-dichlorination, with diastereoselectivity rating of >95:05 and an enantioselectivity rating of 76:24 when diselenide catalyst **AT** was used. Similar results were obtained when using catalyst **J**, which afforded the target product the same diastereoselectivity and enantioselectivity rating of 74:26 (Scheme 39).

**Scheme 40:**
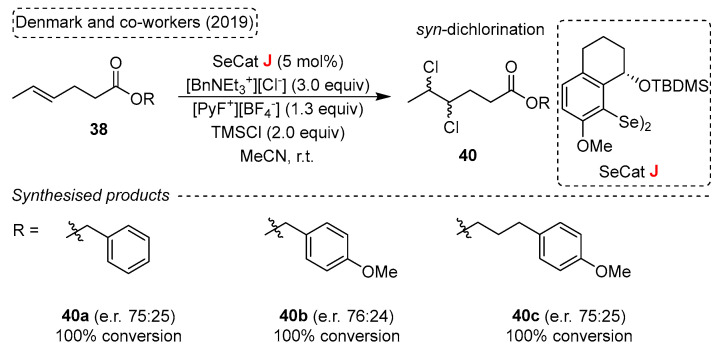
Synthesis of compound **40** via the *syn*-dichlorination of alkenes [41].

The availability of the developed method to obtain different products was limited. Only two different alkenes were applied in the *syn*-dichlorination reaction, using diselenide **J** as the catalyst. In both cases, the obtained products, **40b** and **40c**, showed an enantioselectivity ratio similar to compound **40a** (previously optimised) (Scheme 40).

**Scheme 41:**
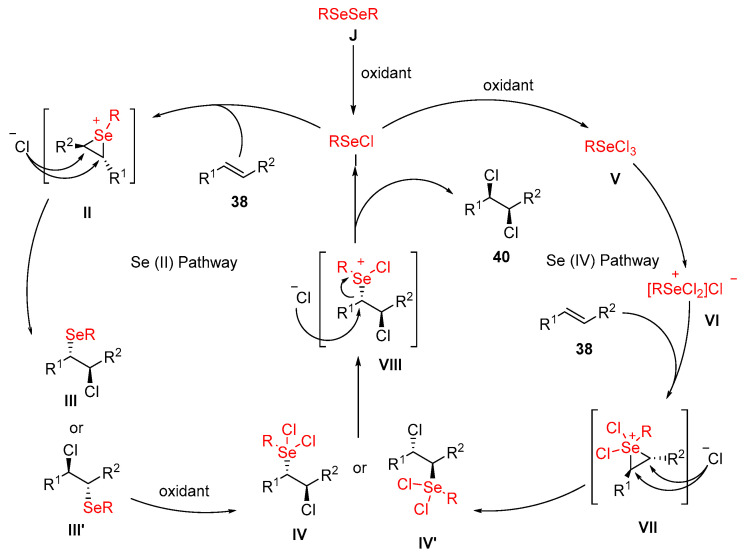
Mechanism proposed for the synthesis of compound **40** via the *syn*-dichlorination of alkene.

The authors proposed a mechanistic pathway based on previous works. In the selenium (IV) path, firstly, diorganyl diselenide **J** is oxidised twice, reaching organylselenyl trichloride **V**, which reacts with olefin **38**, producing seleniranium ion **VII**. The isomers **IV** and **IV’** are produced when seleniranium ion **VII** is opened antarafacially through the reaction with chloride at either of the carbon atoms. The ionisation of the isomers leads to intermediate **VII**, which reacts with chloride through an S_N_2 reaction, affording the final product, **40**, and regenerating intermediate **I**. Moreover, the selenium (II) path starts with the reaction of PhSeCl **I** with alkene **38**, generating seleniranium ion **II**. The nucleophilic addition of chloride to seleniranium ion **II** generates the intermediates **III** and **III’**, which are subsequently oxidised to the intermediates **IV** and **IV’**. The final steps to obtain the desired product, **40**, are the same as the previous path (Scheme 41).

**Scheme 42:**
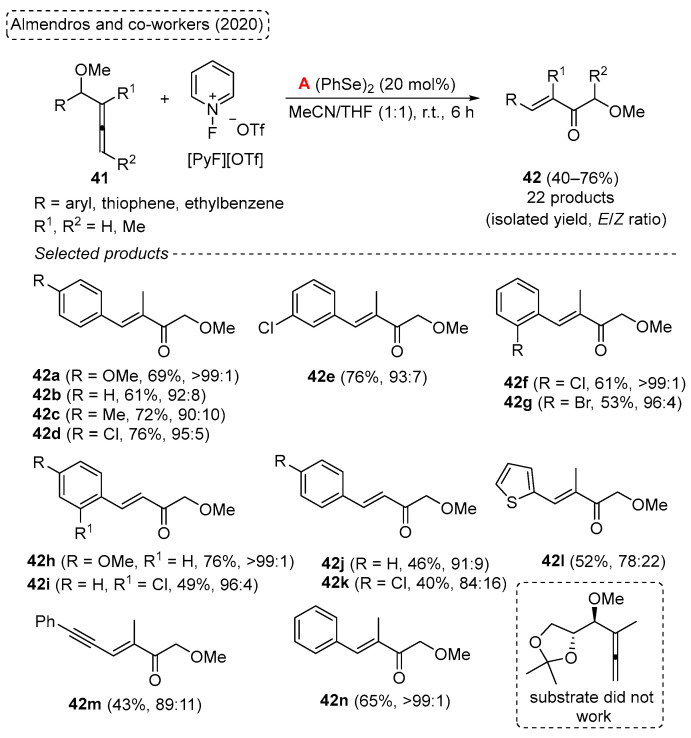
Synthesis of α’-alkoxy enone **42** compounds [42].

The synthesis of α’-alkoxy enone **42** starting from allenyl ether **41** was enabled by oxygenated rearrangement, which is promoted through an organoselenium-based *π*-acid-type catalysis [42]. In this method, 20 mol% of diphenyl diselenide **A** was utilised as the catalyst in a mixture of MeCN and THF as solvents at r.t. In addition, to prove the versatility of the protocol, various allenyl ethers, **41**, were applied under the optimal conditions, obtaining 22 compounds of **42**, with yields ranging from 40% to 76% and good (*E*)-diastereoselectivity (Scheme 42).

**Scheme 43:**
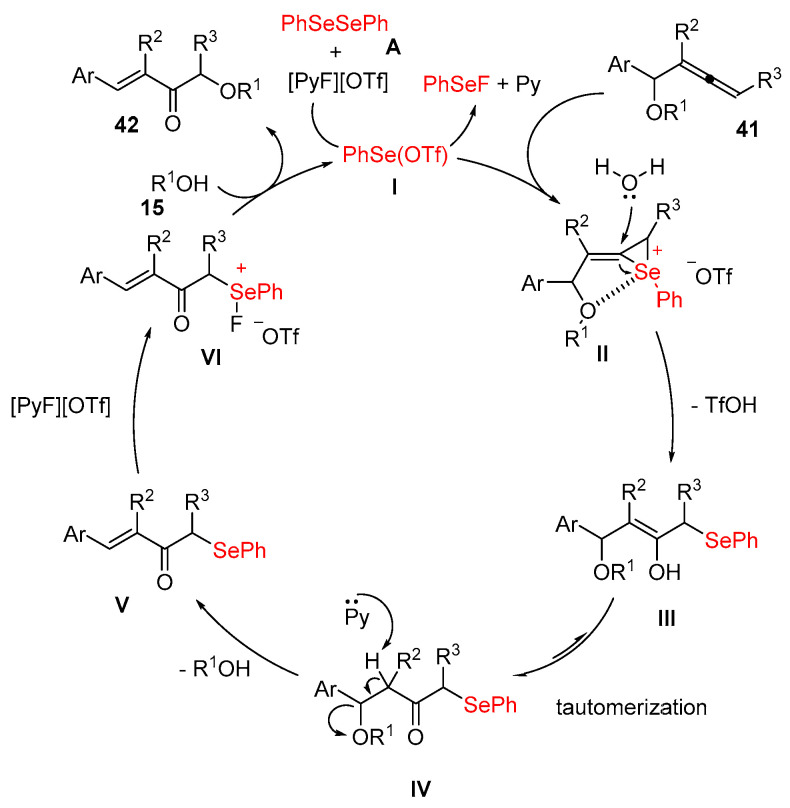
Proposed mechanism for the synthesis of α’-alkoxy enone **42** compounds.

In addition, the authors proposed a mechanism for the developed method. Firstly, diphenyl diselenide **A** is converted into selenium species PhSe(OTf) **I** using the oxidising agent [PyF][OTf]. After that, a regioselective carbophilic reaction with the terminal allene **41** double bond results in the formation of intermediate alkenyl seleniranium **II**, which is in *syn* conformation due to stabilisation by Se–O noncovalent interaction. Subsequently, the nucleophilic addition of the water generates intermediate **III**, which is in equilibrium with the more stable tautomer **IV**. Next, the abstraction of the acidic hydrogen, which promotes the elimination of alcohol **15** and the production of selenonium **V**, is performed by the free pyridine in the medium. Further, selenonium **V** is oxidised by [PyF][OTf], generating selenonium species **VI**. Lastly, the previously generated alcohol **15** reacts with intermediate **VI**, allowing for the formation of the final product, **42**, and the regeneration of the PhSe(OTf) **I** catalyst species (Scheme 43).

**Scheme 44:**
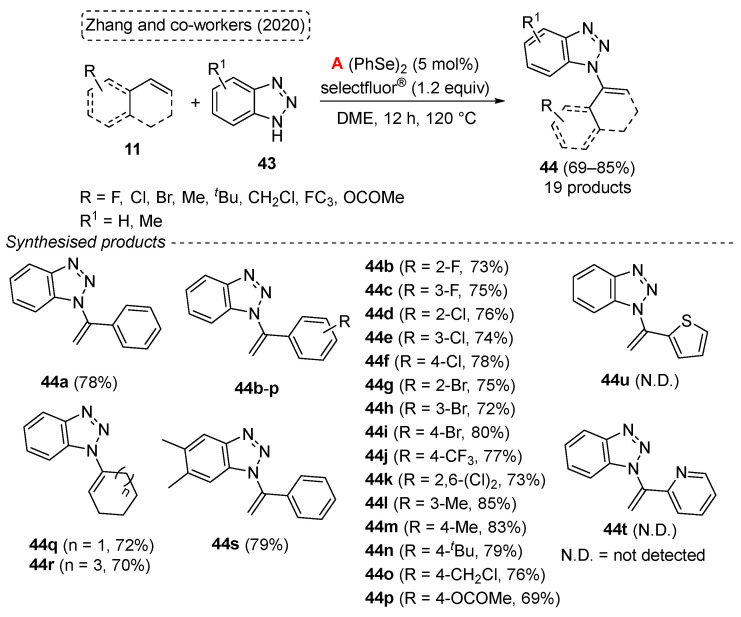
Synthesis of the compounds of **44** via the N^1^-selective *aza*-Wacker reaction [43].

In 2020, Zhang and co-workers [43] proposed a N^1^- and N^2^-selective *aza*-Wacker reaction catalysed by diphenyl diselenide **A**. The method involves the reaction of alkene **11** with benzotriazole **43** for the synthesis of different N^1^- and N^2^-olefinated benzotriazole **44** or **45**. For N^1^-olefinated benzotriazole **44**, the best reaction conditions were shown to be 0.5 mmol of **11** and **43**, 5 mol% of diphenyl diselenide **A**, and 1.2 equiv. of Selectfluor^®^ in dimethyl ether (DME) for 12 h at 120 °C. Under the optimised conditions, 19 target **44** compounds, with yields ranging from 69% to 85%, were obtained. Among the synthesised products, many styrene-based compounds, **11**, containing EDGs and EWGs, were successfully employed. In addition, two cycloalkenes were reacted, generating the products **44q** and **44r** at yields of 72% and 70%, respectively (Scheme 44). After the satisfactory scope obtained by the N^1^-type *aza*-Wacker reaction (compounds **44**), this approach was subsequently employed for the synthesis of N^2^-type *aza*-Wacker compound **45**.

**Scheme 45:**
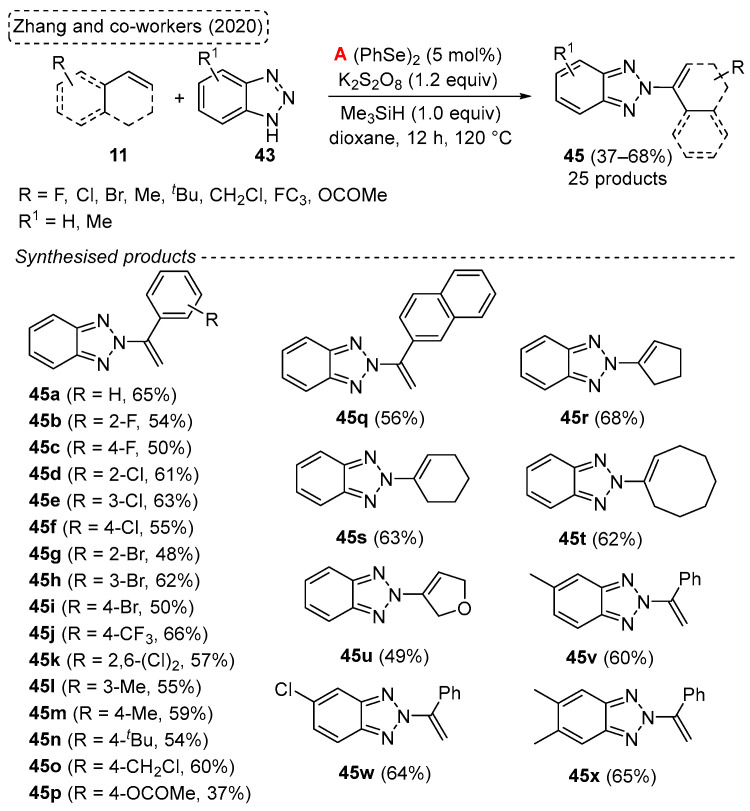
Synthesis of the compounds of **45** via the N^2^-selective *aza*-Wacker reaction [43].

For the synthesis of N^2^-olefinated benzotriazole **45**, the protocol was slightly modified. The best reaction conditions were found to be 0.5 mmol of **11** and **43**, 5 mol% of diphenyl diselenide **A**, 1.2 equiv. of K_2_S_2_O_8_, and 1 equiv. of Me_3_SiH in dioxane for 12 h at 120 °C. Under these conditions, 25 N^2^-olefinated benzotriazole **45** was developed, yielding up to 68%. Styrene **11** containing EDGs and EWGs was successfully employed. In addition, the method was also efficiently applied to inactivated cycloalkene **11** (Scheme 45). These results highlight that not only did the organoselenium-catalysed *aza*-Wacker reaction occur with remarkable regioselectivity, but it also provides a plausible method to substitute the standard metal-catalysed *aza*-Wacker reactions.

**Scheme 46:**
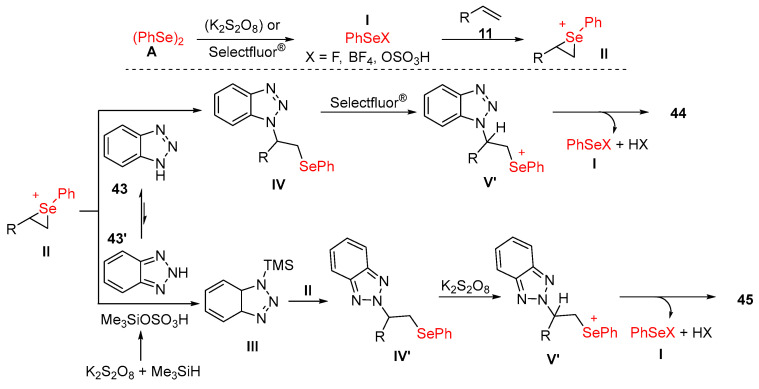
Proposed mechanism for the synthesis of compounds **44** and **45** via the N^1^ and N^2^ -selective *aza*-Wacker reaction.

The authors proposed the following mechanism. The interaction of diphenyl diselenide **A** with K_2_S_2_O_8_ and Selectfluor^®^ results in the formation of a the PhSeX (X = F, BF_4_, and OSO_3_H) **I** species. Extremely reactive PhSeX **I** rapidly interacts with alkene **11** to produce seleniranium ion **II**, followed by the intermolecular nucleophilic addition of benzotriazole **43**, which results in a three-membered ring opening to produce intermediate **IV** (**IV’**). The **IV** (**IV’**) PhSe group is subsequently oxidised to produce intermediate **V** (**V’**). Consequently, proton elimination supported by X (X = F, BF_4_, and OSO_3_H) and selenium moiety removal yield the appropriate compounds, **44** and **45**, and regenerate the PhSeX **I** species. The preliminary steps of the N^2^-selective *aza*-Wacker reaction occur amongst the interactions of benzotriazole, K_2_S_2_O_8_, and Me_3_SiH, generating silylated benzotriazole **III**. This step is followed by an N^2^-type nucleophilic addition on the three-membered seleniranium ion **II**, resulting in the N^2^-selective selenoamination product **45** (Scheme 46).

Zeng and co-workers [44] described an electro-selenocatalytic approach for the hydroazolylation of alkene **46** with azole **43** in the absence of an external oxidant and with minimal catalyst loadings. In this work, the optimal reaction conditions were 0.3 mmol of azole **43**, 0.6 mmol of **46**, diphenyl diselenide **A** as the catalyst (2 mol%), and LiClO_4_ as the electrolyte (1 equiv.) in a mixture of DCE:MeCN:*N*,*N*-dimethylformamide (DMF) (9:0.9:0.1) as the solvent. Additionally, Pt net electrodes (1.0 cm × 2.0 cm) were used in an undivided cell pattern.

**Scheme 47:**
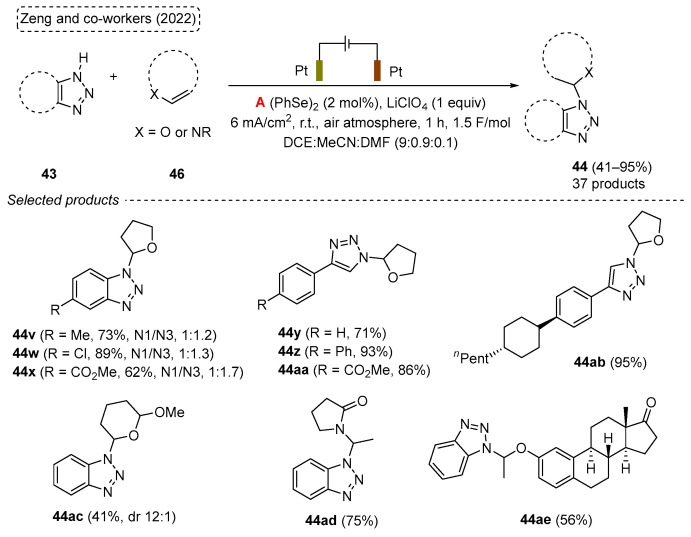
Synthesis of the compounds of **44** [44].

Under the optimised conditions, 37 target compounds of **44** with yields ranging from 41% to 95% were obtained. The reaction was successfully employed in the intermolecular hydroazolylation of electron-rich alkene **46** with azole **43**, using vinyl ethers, enamides, and enamine. Moreover, the proposed approach is suitable for the economical synthesis of pharmaceutically significant azolyl **44** hemiaminal ethers and aminals in reasonable to exceptional yields (Scheme 47).

Further, the notable advantages of this electro-selenocatalytic technique include minimal catalyst loads, external oxidant-free conditions, high current rates and regioselectivities, and great functional group tolerance, as proven by the azolylation of natural product-derived alkenes.

### 2.3. Oxidation and Reduction Reactions

Diorganyl diselenides are well known for having oxidising behaviour. In this section, the oxidation of alcohols and aldehydes, among others, and redox condensation reactions are shown. Usually, the organoselenium catalysts act together with some selective terminal oxidants, such as hydrogen peroxide or atmospheric oxygen. In addition to the dibenzyl diselenide, as well as the most frequently used diphenyl diselenide catalyst, heterogeneous catalysts containing selenium in their structure were used in the studies described in this section.

**Scheme 48:**
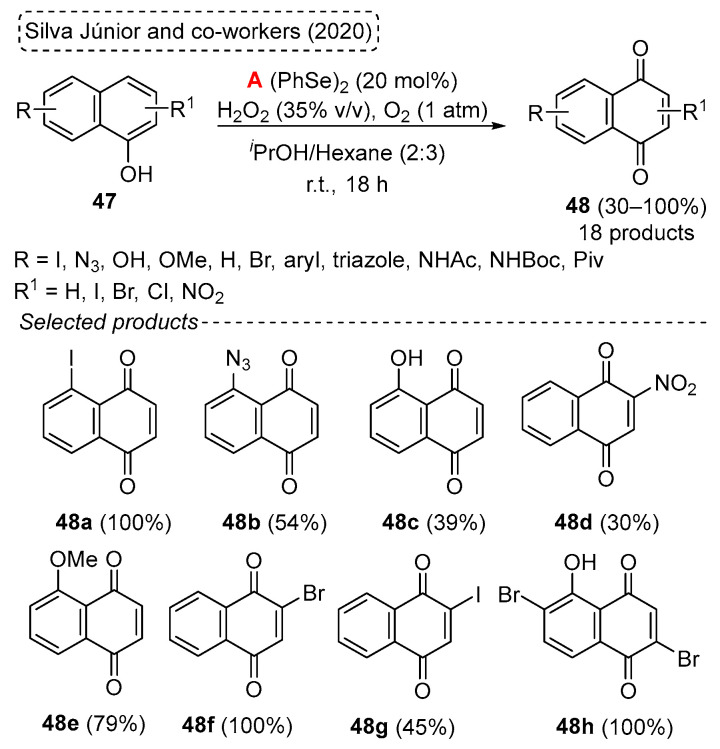
Synthesis of quinone **48** [45].

In 2018, da Silva Júnior and co-workers [45] developed the synthesis of trypanocidal quinone **48** through the oxidation of naphthol **47** using organoselenium **A** and hydrogen peroxide (as a cheap and easy-to-handle oxidising agent). Initially, the authors studied the oxidation with three different diselenides, studying diphenyl diselenide substituted with EDGs [bis(*p*-methoxyphenyl)selenide **W**] or EWGs [bis(*p*-chlorophenyl) diselenide **N**]. In the preliminary studies, diphenyl diselenide showed the best results, obtaining the oxidation of 5-iodonaphthalen-1-ol **48a** at a 100% yield. Under the optimised conditions, 18 products of **48** were obtained, with yields ranging from 30% to 100%. Various functional groups were suitable for the developed reaction, showing the selectivity of the method (Scheme 48).

**Scheme 49:**
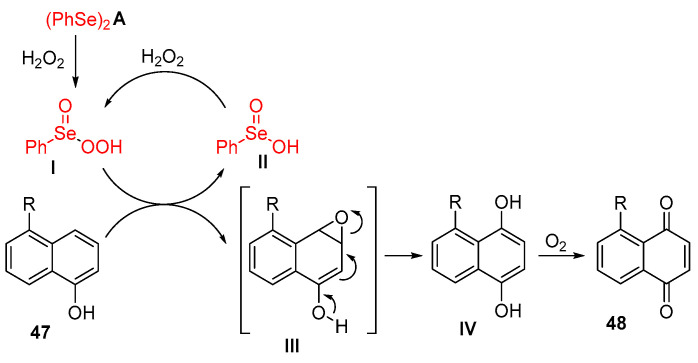
Proposed mechanism for the synthesis of quinone **48**.

The mechanism proposed by the authors starts with the oxidation of diphenyl diselenide **A**, producing benzeneseleninoperoxoic acid **I**. Subsequently, the reaction of benzeneseleninoperoxoic acid **I** with naphthol **47** generates epoxide **III**, which undergoes hydrogen abstraction to produce intermediate **IV**. Lastly, the oxidation of intermediate **IV** generates the final product, **48** (Scheme 49).

**Scheme 50:**
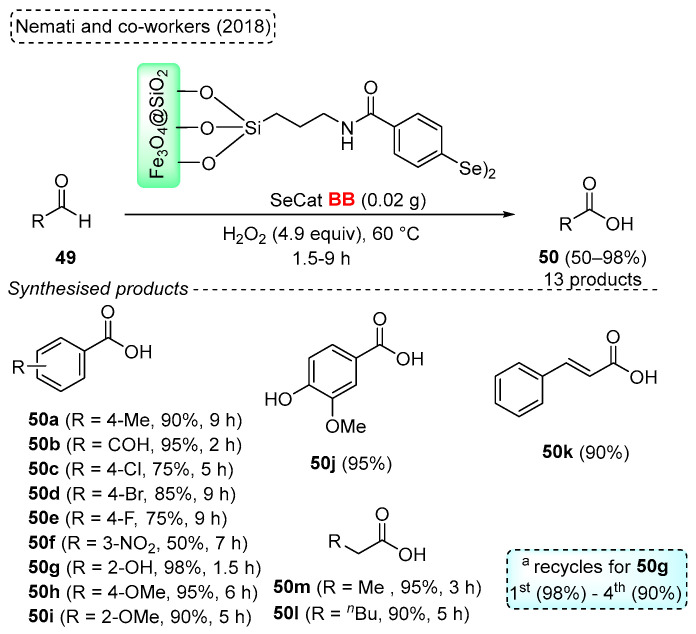
Synthesis of carboxylic acid **50** [46].

Diselenide supported on silica-coated Fe_3_O_4_ magnetic nanoparticles was devised, produced, and presented in the work of Nemati [46] as a new and magnetically recoverable heterogeneous catalyst, **BB**. Moreover, the efficiency of the developed catalyst was studied in the oxidation of aldehyde **49** to carboxylic acid **50** in an aqueous medium. As the best reaction conditions for 1 mmol of aldehyde **49**, 20 mg of the catalyst **BB** and 4.9 equiv. of H_2_O_2_ were used at 60 °C for 1.5 h. Aldehyde **49**, containing both EDGs and EWGs, was suitable for oxidation; however, those containing EDGs showed better results. In addition, aldehyde **49** (cinnamaldehyde and those containing an aliphatic chain) was effectively oxidised and yielded the respective carboxylic acid, **50**, in high yields (Scheme 50).

**Scheme 51:**
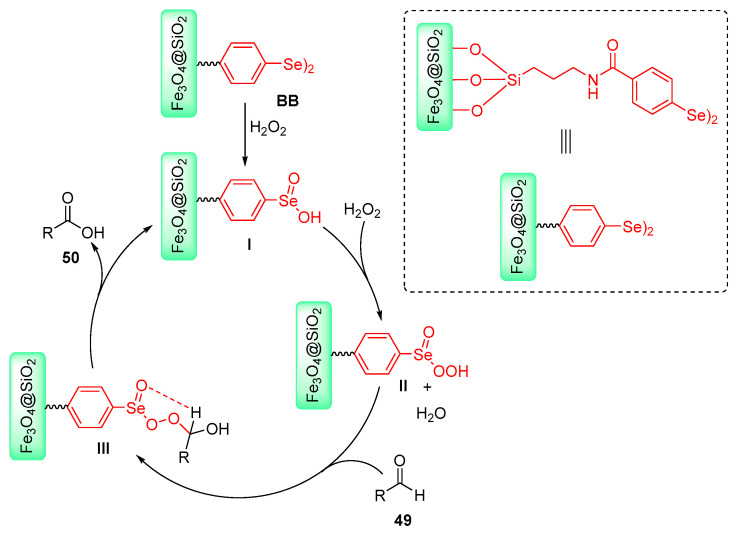
Proposed mechanism for the synthesis of carboxylic acid **50**.

In the first step of the mechanism, the oxidation of catalyst **BB** by H_2_O_2_ generates seleninic acid **I**. Afterwards, H_2_O_2_ oxidises seleninic acid **I,** promoting the formation of peroxyseleninic acid **II,** which reacts with aldehyde **49** and generates intermediate **III**. After the intramolecular rearrangement promoted by the H bond interaction of intermediate **III**, product **50** is released and seleninic acid **I** is renewed for the catalytic cycle (Scheme 51).

In 2018, Yang and co-workers [47] developed the oxidation of alkene **11** with trimethylsilyl azide (TMSN_3_) **51** in air at r.t., resulting in a new and simple visible-light-enabled approach for the synthesis of α-azidoketone **52**. Using cheap and low-toxic Rose Bengal (RB) (1 mol%) and diphenyl diselenide (5 mol%) **A** as co-catalysts, a series of α-azidoketones, **52**, could be quickly and effectively produced in moderate to good yields by cascade C–N and C=O bond formation.

**Scheme 52:**
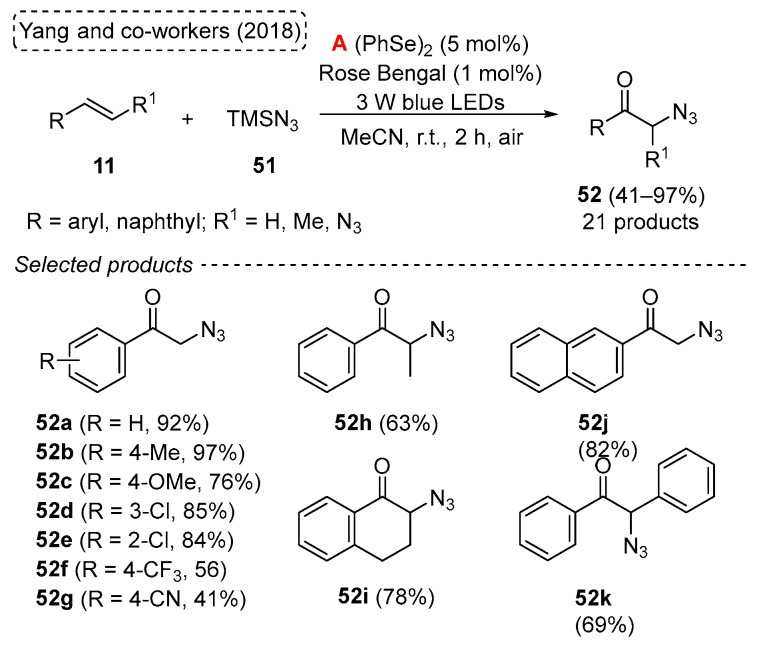
Synthesis of α-azidoketone **52** [47].

With this method in hand, the authors were able to synthesise 21 compounds of **52**, with yields ranging from 41% to 97% (Scheme 52). When using this procedure, aromatic alkene **11** with either EDGs or EWGs on the aryl rings proved to be appropriate for the reaction. The substrates’ electronic effect has a significant impact on how efficiently the reaction proceeds and those bearing substituted aromatic alkenes with strong EDGs gave the target product at a better yield than those with strong EWGs. In addition, different functional groups and substrates containing steric hindrance were suitable for the reaction. However, none of the expected products were obtained when aliphatic alkenes, like hex-1-ene and cyclopentene, were employed as substrates.

**Scheme 53:**
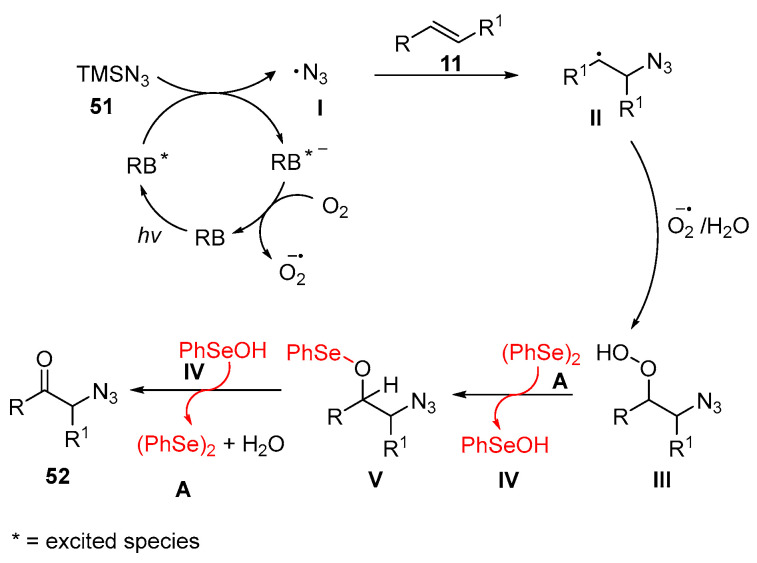
Proposed mechanism for the synthesis of α-azidoketone **52**.

The mechanism proposed by the authors is shown in Scheme 53. Initially, RB was used to create excited RB^*^ under the influence of visible light. After that, TMSN_3_ **51** and RB^*^ underwent a SET reaction, resulting in the formation of azido radical **I** and RB^•−^ radical anions. Moreover, the oxidation of RB^•−^ by molecular oxygen (air) would result in the formation of the ground state RB and O_2_^•−^. Alkene **11** was then combined with azido radical **I** to create alkyl radical **II**. Subsequently, hydroperoxide intermediate **III** was produced by the reaction of radical **II** with O_2_^•−^ and H_2_O. Reactive organoselenium species **V** and PhSeOH **IV** were produced when hydroperoxide **III** oxidised diphenyl diselenide **A**. The target product, **52**, was finally formed through the reaction of PhSeOH **IV** with reactive intermediate **V** while also regenerating diphenyl diselenide **A** and eliminating water (Scheme 53).

**Scheme 54:**
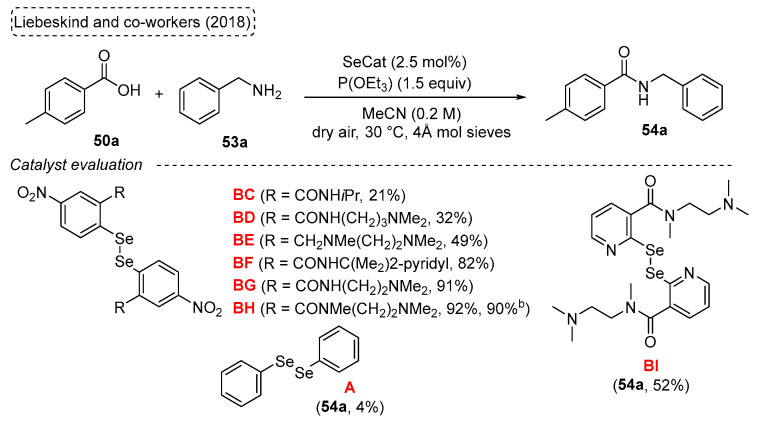
Catalyst evaluation for the synthesis of compound **54a** [48].

Liebeskind and co-workers [48] proposed an easy-to-perform organocatalytic process in 2018, which relies on the selenols’ capacity to react with oxygen in air directly without the use of metal catalysts. The redox dehydration of *p*-toluic acid **50a** and benzylamine **53a** was first evaluated by employing various diselenides as catalysts at 2.5 mol% loadings at 30 °C in MeCN (Scheme 54). *Ortho* amidic-substituted aryl diselenides **BF**, **BG**, and **BH** were shown to be the most efficient catalysts for the proposed reaction (formation compound **54a**). Thus, the location of the attached NMe_2_ unit in relation to the amidic N atom points to the major contribution of intramolecular geometric variables to this catalysis, changing the reactivity of diselenide.

**Scheme 55:**
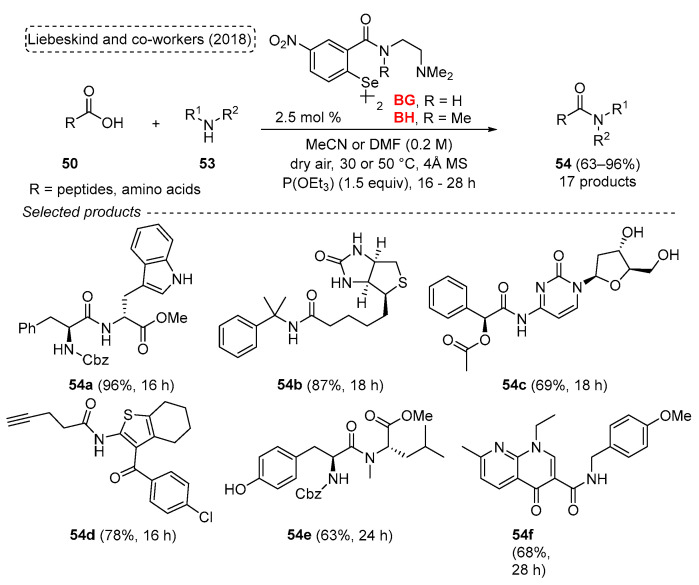
Synthesis of the amidic and peptidic compounds of **54** [48].

For the best reactional conditions, the authors found 2.5 mol% of diselenides **BG** or **BH** in MeCN or DMF at 30 °C or 50 °C, using 1.5 equiv. of triethyl phosphite as a terminal reductant for 16 to 28 h in reaction with the starting materials **50** with **53** was optimal. Under these conditions, the authors developed a wide range of amidic and peptidic compounds, **54** (17 products), with yields ranging from 63% to 96%. In general, the authors developed a method with simple aerobic conditions, a properly designed diselenide, and low loadings of organocatalytic oxidants for application in the synthesis of interesting amides and peptides **54** (Scheme 55).

**Scheme 56:**
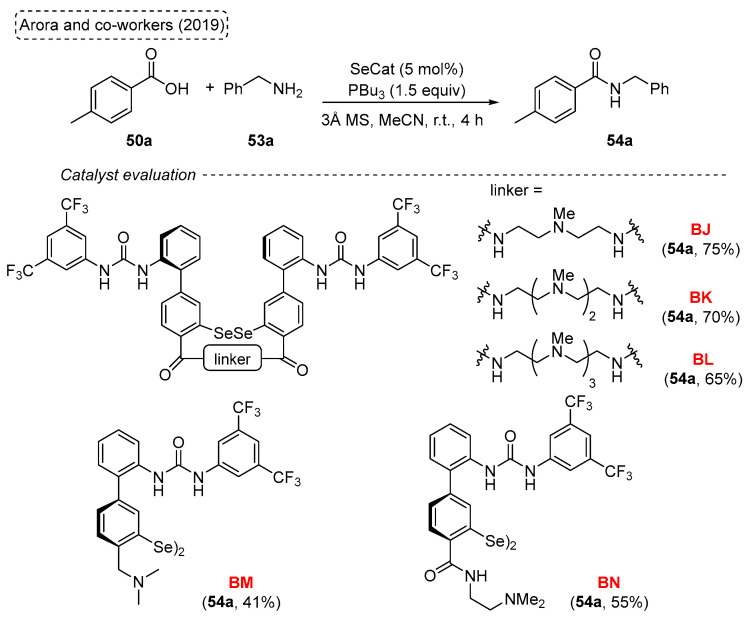
Catalyst evaluation for the synthesis of compound **54a** [49].

In the next year (2019), a similar method was reported by Arora and co-workers [49], which proposed the synthesis of dipeptides using organoselenium catalysis. In this work, redox condensation promotes the formation of selenoester, activating and promoting the reaction on the carboxylic acid. The authors started with a preliminary screening of different organoselenium catalysts: **BJ**–**BN**, all containing a urea and a tertiary amine portion. The first reactions were carried out with *p*-toluic acid **50a**, 2 equiv. of benzylamine **53a**, 5 mol% of Se catalyst **BJ**–**BN**, 1.5 equiv. of tributylphosphine, and 3 Å MS in acetonitrile at r.t. Among the five developed catalysts, the best result was shown when **BJ** was used, which generated the final product, **54a**, at a 75% yield (Scheme 56).

**Scheme 57:**
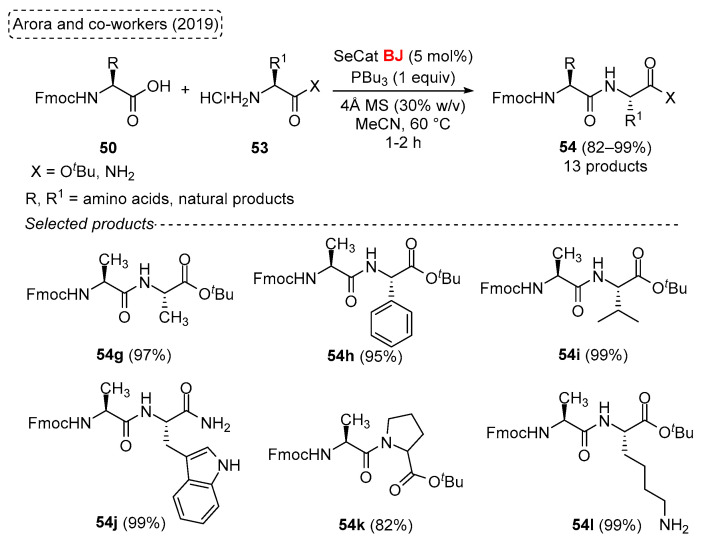
Synthesis of dipeptides **54** [49].

After choosing the best selenium catalyst (**BJ**), the versatility of the protocol was evaluated with various commercially available fluorenylmethyloxycarbonyl (Fmoc) amino acids, **50**, in the reaction with starting material **53** (Scheme 57). Dipeptide **54**, containing alanine, tryptophan, lysine, valine, and a proline moiety, was obtained in high yields using this protocol: 13 products with a yield ranging from 82% to 99%. In addition, the synthesis of an oligopeptide was developed in the solid phase by employing a Se catalyst (**BJ**), demonstrating the potential of this to react within more than one amine bond upon reaction.

**Scheme 58:**
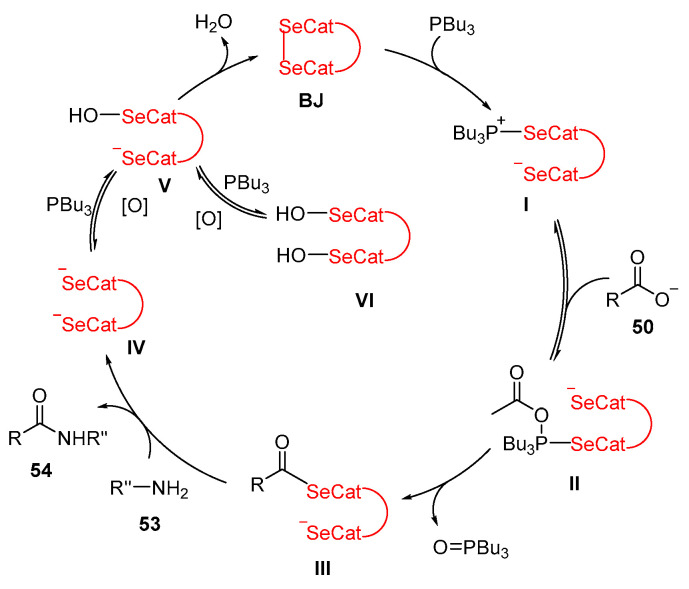
Proposed mechanism for the synthesis of dipeptide **54**.

Lastly, based on the obtained results, a mechanism was proposed by the authors (Scheme 58). In the first step, selenophosphonium **I** is generated via the reduction of the Se catalyst (**BJ**) with tributylphosphine. Selenophosphonium **I** easily associates with carboxylate **50**, producing intermediate **II**, which undergoes an intramolecular acyl transfer, leading to selenoester **III**. The consecutive aminolysis of selenoester **III** generates the final product, **54**, plus bis-selenolate **IV**, which can be oxidised to recover the Se catalyst **BJ**.

**Scheme 59:**
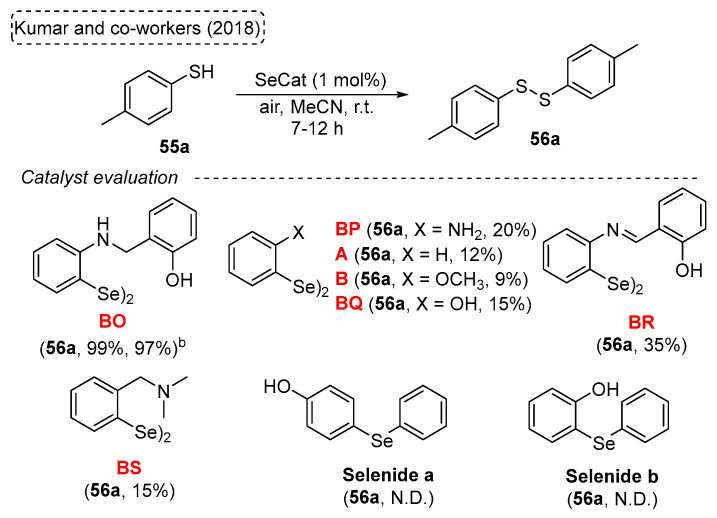
Catalyst evaluation for the oxidation of organothiol **55a** to diorganyl disulphide **56a** [50].

In 2018, Kumar and co-workers [50], proposed an aerial oxidation of organothiols **55** to diorganyl disulphide **56**, catalysed by diorganyl diselenides. Firstly, the authors evaluated different species of catalyst through the aerial oxidation of *p*-toluenethiol **55a** to 1,2-di-*p*-tolyl disulphide **56a** in acetonitrile at r.t. Diorganyl diselenides containing various functional groups were tested; however, the **BO** catalyst was the most effective for the reaction, providing a 97% yield for the desired product, **56a** (Scheme 59).

**Scheme 60:**
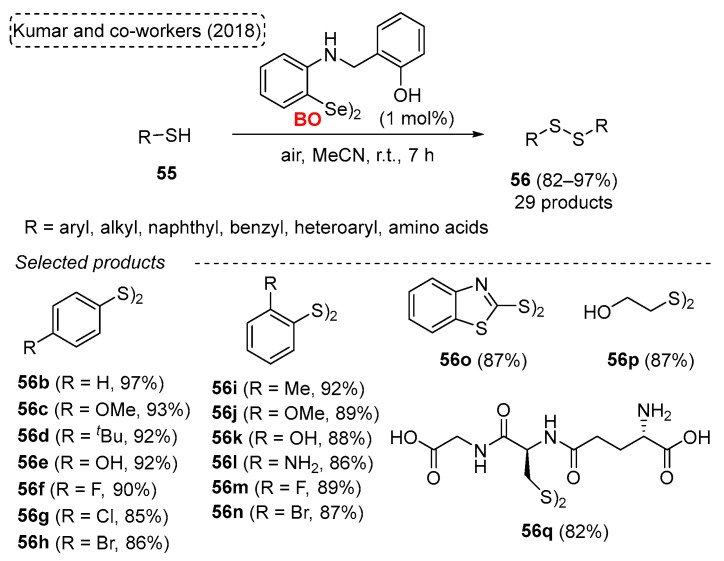
Oxidation reaction of organothiol **55** to diorganyl disulphide **56** [50].

Furthermore, the aerial oxidation of several organothiol **55** compounds was subsequent evaluated in the presence of 1 mol% of the enzyme mimic **BO** (selenium catalyst). By using the developed method, 29 compounds of **56** were obtained, with a yield ranging from 82% to 97% (Scheme 60). Thiols **55**, containing different functional groups, such as alcohol, amine, methoxy, and halogen, was successfully employed in the reaction. In addition, the authors were able to synthesise disulphide by starting from biologically relevant thiols for compound **56q** bearing the glutathione moiety.

**Scheme 61:**
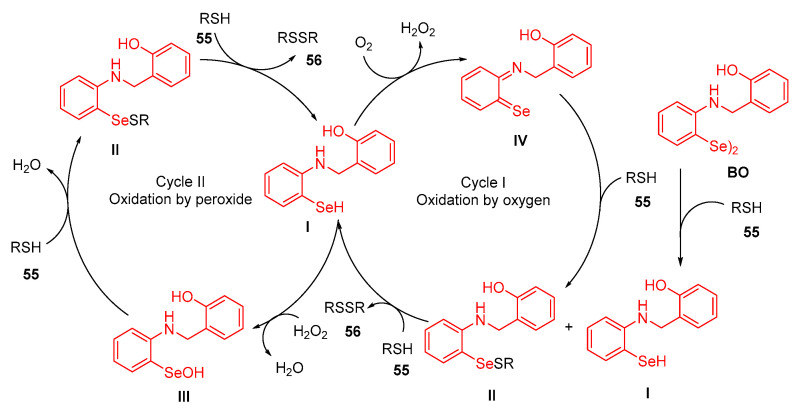
Proposed mechanism for the oxidation of organothiol **55** to diorganyl disulphide **56**.

Lastly, the authors proposed the mechanism as follows (Scheme 61). Selenenyl sulphide **II** and selenol **I** are produced when diorganyl diselenide **BO** interacts with one equivalent of thiol **55**. Selenenyl sulphide **II** may then combine with another thiol **55** molecule to form selenol **I**, as was estimated. By oxidising selenol **I** with oxygen, selone **IV** and hydrogen peroxide are created by proton and electron transfer from N– and Se–H bonds (Cycle I). Selenol and the radicals HO_2_• and PhS• might also be formed when selenium sulphide **II** reacts with oxygen; the second radical then dimerises to form RSSR **56**. The catalytic cycle is completed by a proton transfer, which results from the production of selenenyl sulphide **II** by the nucleophilic addition of sulphur from RSH **55** to the selenium in selone **IV**. In cycle II, selenol **I** is oxidised by peroxide, generating selenenic acid **III**, which is then reduced by a molecule of RSH **55** to produce water and selenenyl sulphide **II**, which reacts with a second molecule of RSH **55**, producing the expected disulphide **56**.

**Scheme 62:**
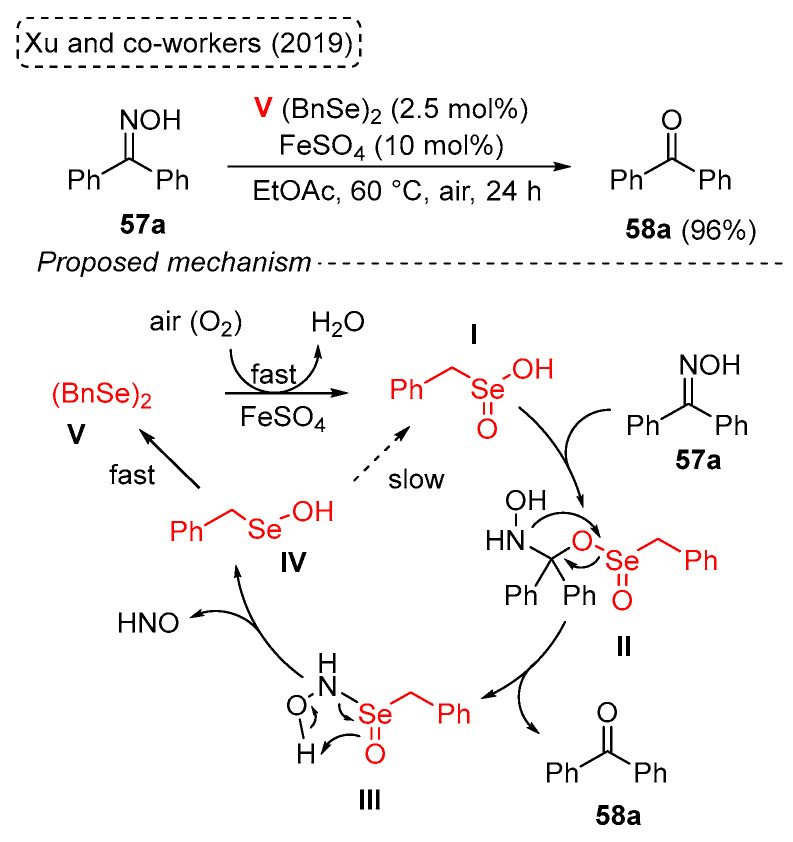
Synthesis and proposed mechanism for the preparation of ketone **58a** [51].

Xu and co-workers [51] developed a mild and green oxidative deoximation of ketoxime **57a** through organoselenium catalyst **V** and using FeSO_4_ as a co-catalyst. For the best reaction conditions, the authors found that 1 mmol of ketoxime **57a**, 2.5 mol% of benzyl diselenide **V** and 10 mol% of FeSO_4_ as catalysts in ethyl acetate as solvent for 24 h at 60 °C was optimal. Under these conditions, the final product, **58a**, was obtained at a 96% yield. Moreover, a possible mechanism was proposed whereby FeSO_4_ initially promotes the oxidation of the diselenide **V**, forming seleninic acid **I**, which goes through nucleophilic addition with oxime **57a**, generating intermediate **II**. After a rearrangement, product **58a** is formed, and organoselenium intermediate **III** is released. Following the reduction of intermediate **III**, a reduced species, PhCH_2_SeOH **IV**, and hyponitrous acid (HNO) might be produced. After that, intermediate **IV** is reduced, regenerating the initial diselenide catalyst **V** (Scheme 62). This study is noteworthy primarily because it uses an aliphatic diselenide, and the synthesised product was isolated at a high yield.

**Scheme 63:**
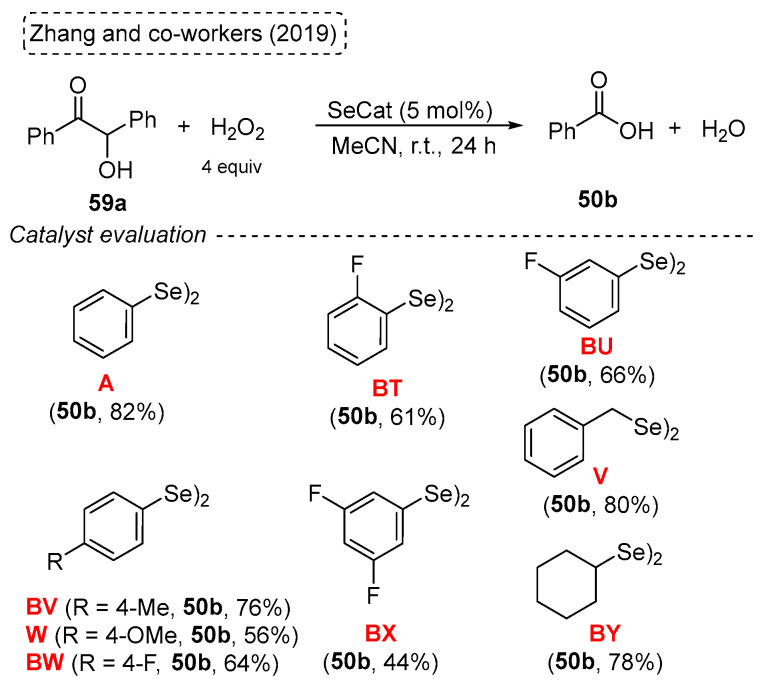
Catalyst evaluation for the synthesis of carboxylic acid **50b** [52].

The oxidative degradation of benzoin **59** to benzoic acid **50**, catalysed by diselenide species, was studied by Zhang in 2019 [52]. At the beginning of the work, the reaction was carried out with 1 mmol of **59a**, 4 equiv. of hydrogen peroxide, and 5 mol% of Se catalyst in acetonitrile as the solvent for 24 h at r.t. After the screening of the organoselenium catalysts, diphenyl diselenide **A** showed the best performance when compared to the others, yielding the desired benzoic acid **50b** at 82% (Scheme 63).

**Scheme 64:**
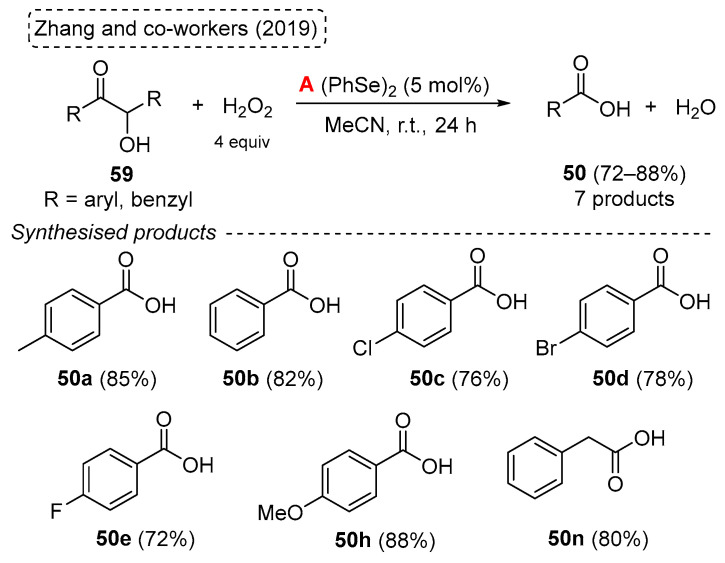
Synthesis of carboxylic acid **50** [52].

Under optimal conditions, a variety of benzoin derivatives of **59** were applied as substrates for the reaction. The method yielded the appropriate carboxylic acid **50**, producing seven products, with yields ranging from 72% to 78%. Overall, the compounds containing EDGs (**50a**: *p*-Me, 85% and **50h**: *p*-OMe, 88%) were slightly more effective in the reaction compared to the ones containing EWGs (**50c**: *p*-Cl, 76%; **50d**: *p*-Br, 78%, and **50e**: *p*-F,72%) (Scheme 64).

**Scheme 65:**
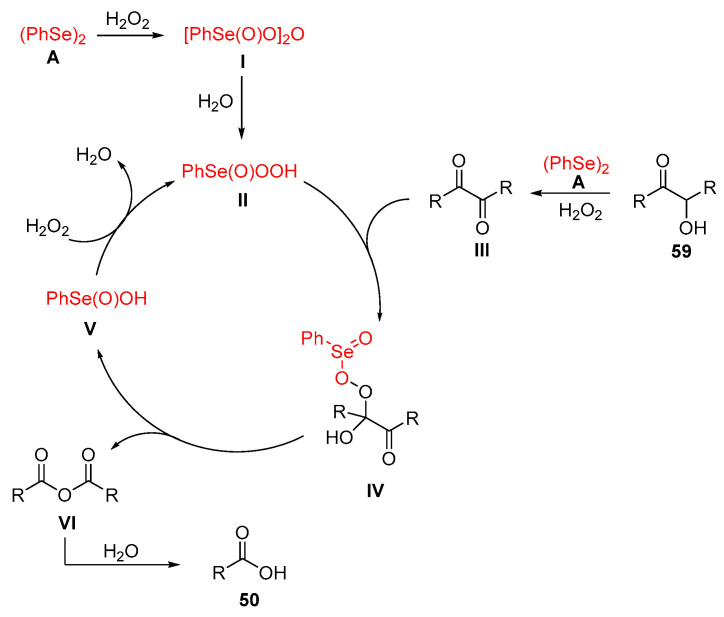
Proposed mechanism for the synthesis of carboxylic acid **50**.

After some control experiments, the following mechanism was proposed (Scheme 65). When diphenyl diselenide **A** is first oxidised with H_2_O_2_, it produces peroxide **I**, which then produces organoselenium species **II**. H_2_O_2_ easily converts benzoin **59** to compound **III** in the presence of the diphenyl diselenide **A** catalyst. Oganoselenium intermediate **IV** is produced by the nucleophilic addition of **II** to a carbonyl group of **III**. Following a C–C bond cleavage reaction in intermediate **IV**, benzoic anhydride **VI** and benzeneseleninic acid **V** are produced. While the benzoic anhydride **VI** is hydrated and generates the desired product, **50**, benzeneseleninic acid **V** is oxidised, promoting the regeneration of **II** of the catalytic cycle.

**Scheme 66:**
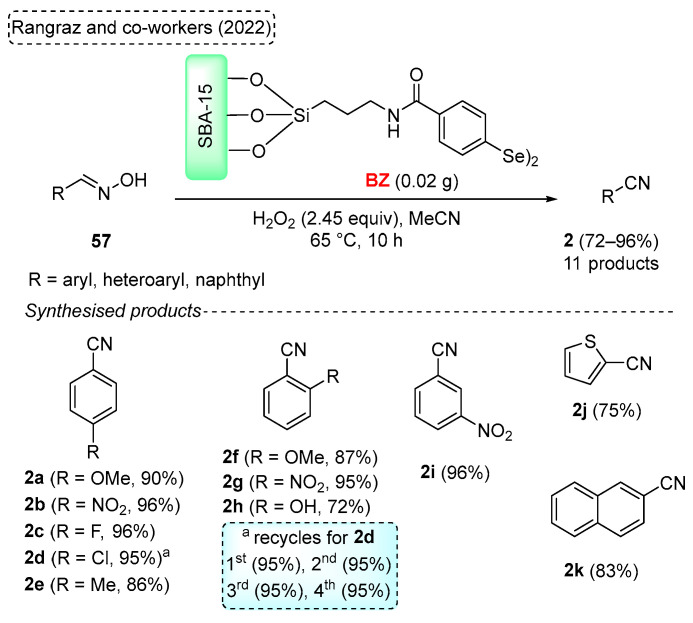
Synthesis of nitrile **2** [53].

Rangraz and co-workers [53] promoted the oxidation of oxime **57** to nitrile **2** by employing a new selenium-based catalyst. In order to create the novel organoselenium functionalised SBA-15 **BZ**, diphenyl diselenide **A** was immobilised on Santa Barbara Amorphous-15 (SBA-15), which was previously functionalised with (3-aminopropyl) triethoxysilane. After the synthesis of catalyst **BZ**, the authors found (as the best reaction) the conditions of 1 mmol of oxime **57**, 0.02 g of catalyst **BZ**, and 2.45 equiv. of hydrogen peroxide in acetonitrile for 10 h at 65 °C to be optimal. With the optimised conditions, 11 compounds of **2** were produced in moderate to high yields using a variety of aldoximes, including substrates with EWGs or EDGs and bulky groups. Furthermore, there was no significant reduction in catalytic activity after using catalyst **BZ** for a minimum of four subsequent cycles (Scheme 66). This is the first report described in this review that uses a heterogeneous catalyst supported on a magnetic material; moreover, it was efficiently used for four recycles in the study carried out by the authors.

**Scheme 67:**
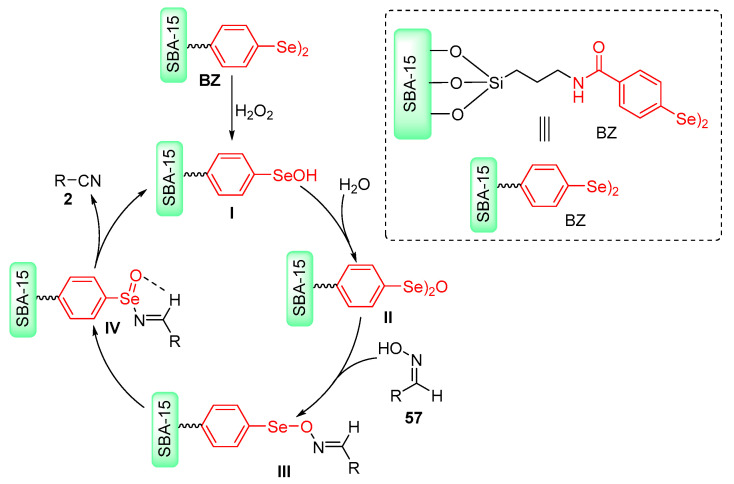
Proposed mechanism for the synthesis of nitrile **2**.

The proposed mechanism begins as the hydrogen peroxide is first used to produce selenenic acid **I**, which is subsequently transformed into the appropriate selenenic anhydride **II**. Afterwards, intermediate **III** is produced through the condensation of aldoxime **57** with selenenic anhydride **II**. Next, intermediate **III** easily converts into the stabilised intermediate **IV**, which passes through a selenoxide *syn*-elimination to regenerate the catalytic species **I** before eliminating nitrile **2** (Scheme 67).

**Scheme 68:**
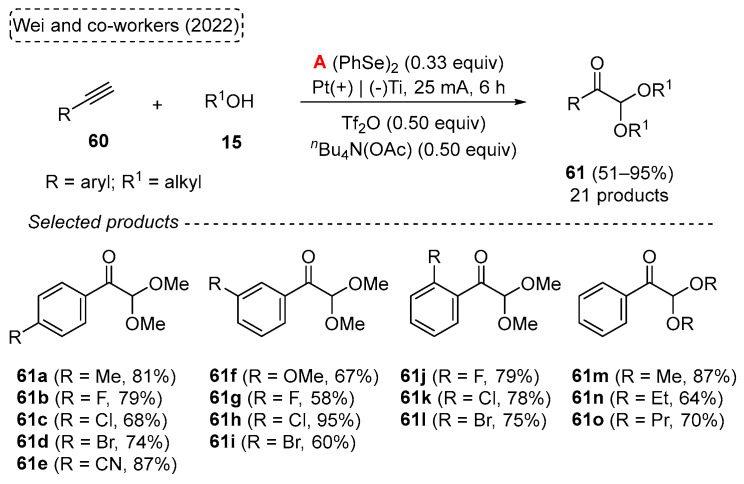
Synthesis of *α*-keto acetal **61** [54].

In 2022, Wei and co-workers [54] developed the synthesis of *α*-keto acetal **61** from the widely accessible terminal alkyne **60** and alcohol **15** by employing an electrochemical method associated with selenium catalyst **A**. As a feature in this work, the effective combination of electrochemical oxidation and organic selenium catalysis easily avoided the need for external chemical oxidants. This dual catalytic system produced a variety of the *α*-keto acetals of **61** with moderate to good yields at r.t. A wide range of the substituted alkynes of **60** and aliphatic alcohols of **15** were suitable for the reaction, reaching 21 compounds of **61** with 51% to 95% yields (Scheme 68).

**Scheme 69:**
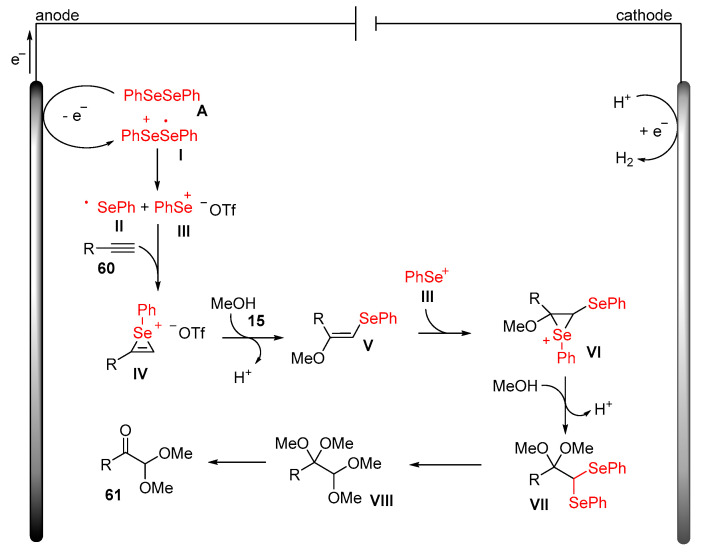
Proposed mechanism for the synthesis of *α*-keto acetal **61**.

The first step of the proposed mechanism is the selenium cation **III** being produced on the anode by a SET from diphenyl diselenide **A**. Subsequently, intermediate **I** goes through a Se–Se bond cleavage, generating radical **II** and electrophilic selenium species **III**. The reaction of electrophilic selenium **III** with alkyne **60** promotes the formation of seleniranium **IV**, which undergoes a nucleophilic addition of alcohol **15**, forming intermediate **V**. Then, intermediate **V** goes through a new sequence of formation with seleniranium **VI**, followed by the nucleophilic addition of alcohol **15**, generating intermediate **VII**. Lastly, a process of alcoholysis is followed by hydrolysis, which leads to the formation of the final product, **61** (Scheme 69).

**Scheme 70:**
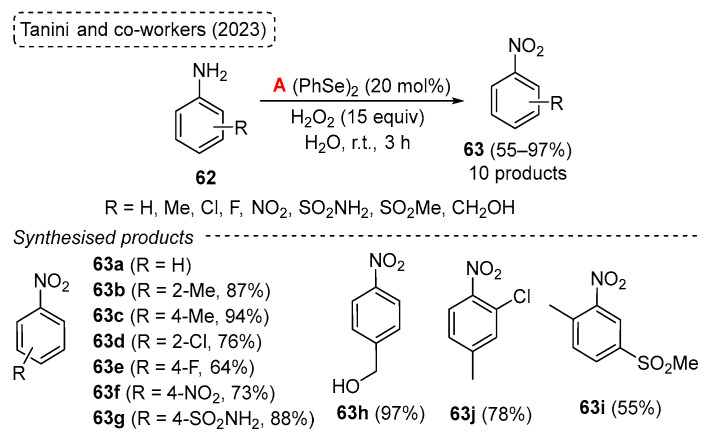
Synthesis of the nitrobenzene compounds of **61** [55].

The synthesis of nitroarene **63** through the oxidation of aniline **62** catalysed by selenium in water, was developed by Tanini and co-workers in 2023 [55]. The developed method employs 20 mol% of diphenyl diselenide **A** as the catalyst and a large amount of H_2_O_2_ (15 equiv.) as the oxidising agent in water for 3 h at r.t. Under the optimised reaction conditions, a variety of the nitroarenes of **63** were readily synthesised, with a yield ranging from 55% to 97% (Scheme 70). The Se-mediated approach worked well to successfully convert aniline **62,** containing both EDG and EWG substituents, with regard to the respective substituted nitro-derivatives of **63**.

**Scheme 71:**
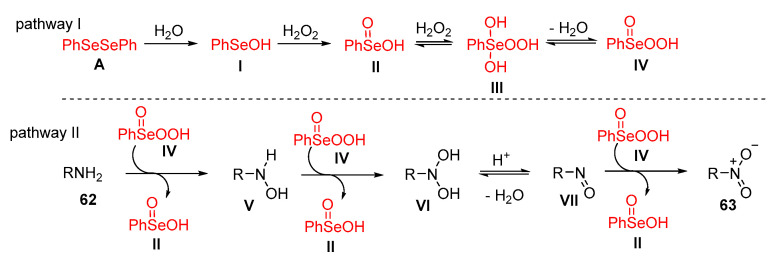
Proposed mechanism for the synthesis of the nitrobenzene compounds of **61**.

The authors proposed the following mechanism. In the first step, hydrogen peroxide is employed for oxidising diphenyl diselenide **A**, generating benzeneselenenic acid **I**, which is subsequently oxidised to benzeneseleninic acid **II** (Scheme 71). Intermediate **II** is further oxidised by H_2_O_2_, generating the expected peroxyselenurane **III**, which suffers a process of dehydration to produce peroxybenzeneseleninic acid **IV**. In the second step of the mechanism, an appropriate reaction of **IV** with aniline **62** generates hydroxylamine **V**, which is subsequently oxidised again by **IV** to yield the dihydroxylamine compound **VI**. After the acid-promoted water elimination of dihydroxylamine **VI**, nitroso intermediate **VII** is obtained, which undergoes oxidation to generate the expected product, **63**.

### 2.4. Reactions Involving Phosphorous-Containing Starting Materials

The organoselenium catalysts are very versatile, with the ability to react with a plethora of organic compounds. Substrates containing the phosphorus atom have been explored under different protocols using small amounts of organoselenium catalysts.

**Scheme 72:**
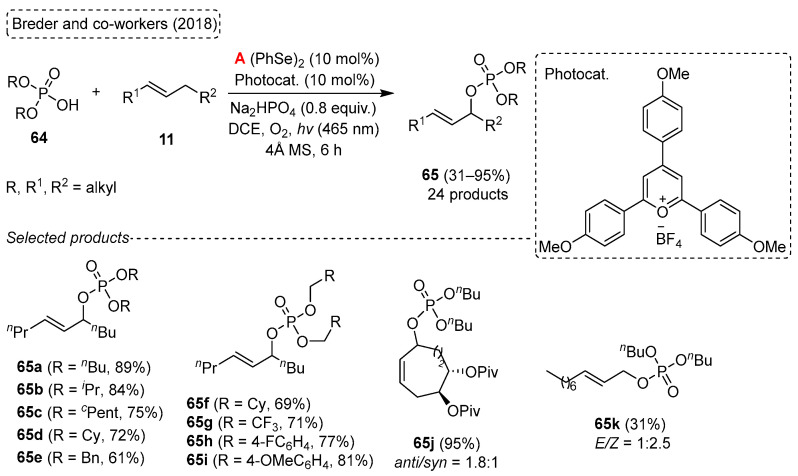
Synthesis of allylic phosphate **65** [56].

Breder and co-workers, in 2018 [56], described the use of diphenyl diselenide **A** as a catalyst to synthesise allylic phosphate **65** by the direct phosphatation of alkene **11** through a single-step protocol, which uses inactivated hydrogen phosphate **64**. In this protocol, a wide range of the dialkyl hydrogen phosphates of **64**, as well as acyclic or cyclic alkene **11**, were permitted when reacted in the presence of 10 mol% of diphenyl diselenide **A** as the catalyst, 10 mol% of a photocatalyst, and 0.8 equiv. of Na_2_HPO_4_ in DCE as the solvent, with O_2_ as the oxidant under *hv* (465 nm) and 4 Å MS for 6 h. Under these conditions, the starting materials **64** and **11** were efficiently reacted, producing the target phosphate products of **65** (24 products) with yields ranging from moderate to excellent (31–95%) (Scheme 72).

**Scheme 73:**
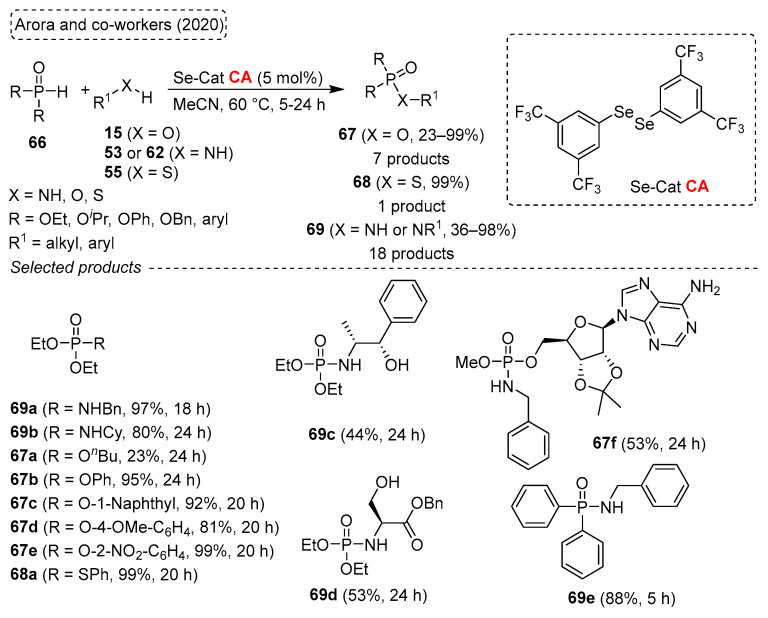
Functionalisation of hydrophosphoryl compounds via the Atherton−Todd reaction [57].

In 2020, Arora and co-workers [57] reported the use of diaryl diselenide **CA** as a catalyst in the Atherton-Todd reaction to perform a functionalisation of hydrophosphoryl compounds through the reaction of starting materials containing phosphorus **66** with nucleophiles **15**, **55**, **53**, or **62**. The authors performed a preliminary screening of different diaryl diselenides, and the best result was obtained when Se-Cat **CA** was used (Scheme 73).

The protocol describes the reaction of phosphine oxide or *H*-phosphonate **66** with amines **53** or **62** in the presence of 5 mol% 1,2-bis(3,5-bis(trifluoromethyl)phenyl) diselenide **CA** as the catalyst and MeCN as the solvent at 60 °C. Under these conditions, several phosphoramidate, phosphonamidate, and phosphinamide **69** compounds were obtained (18) at moderate to excellent yields (36–98%). This approach was extended to phenols or alcohol **15** and thiophenol **55**, but in these cases, it was necessary to use 5 mol% of 4-(dimethylamino)pyridine (DMAP) as the base. When phenols or alcohols **15** or **55** were used as the starting materials, phosphate **67** (7 products) was obtained, whereas when thiophenol **55** was used, the target phosphorothioate **68a** was generated at an excellent yield (99%) (Scheme 73).

**Scheme 74:**
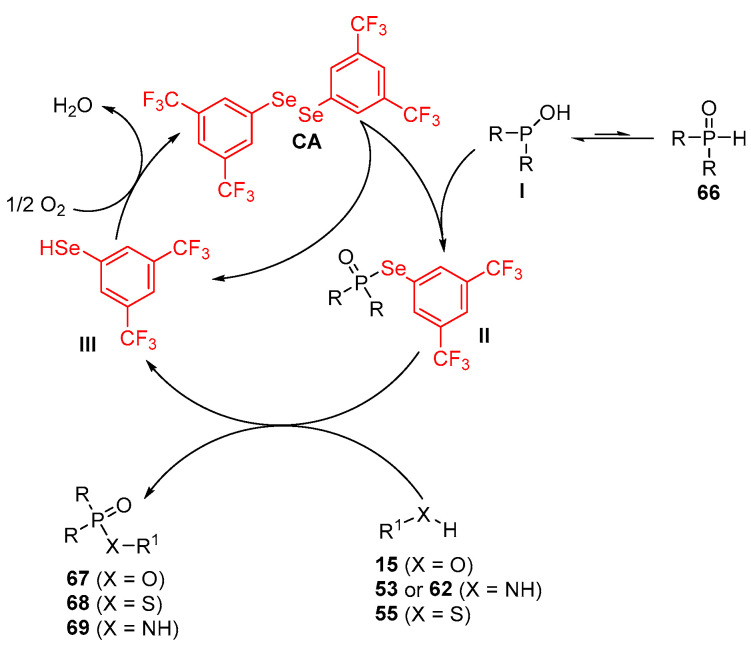
Proposed mechanism for the functionalisation of hydrophosphoryl compounds.

The plausible mechanism described by the authors starts with the equilibrium of R_2_P(O)−H **66** with compound **I**, which reacts with diselenide **CA** to produce R_2_P(O)−Se intermediate **II**, as well as forming arylselenol **III**. Subsequently, phosphoryl selenide intermediate **II** (previously formed) can undergo nucleophilic substitution with amines **53** or **62**, alcohols, phenol **15,** or thiophenol **55** to give the target products **67** (X = O), **68** (X = S), and **69** (X = NH) and a new equivalent of arylselenol **III**, which, in the presence of air, is reoxidised to regenerate the diaryl diselenide **CA** catalyst for a new cycle (Scheme 74).

**Scheme 75:**
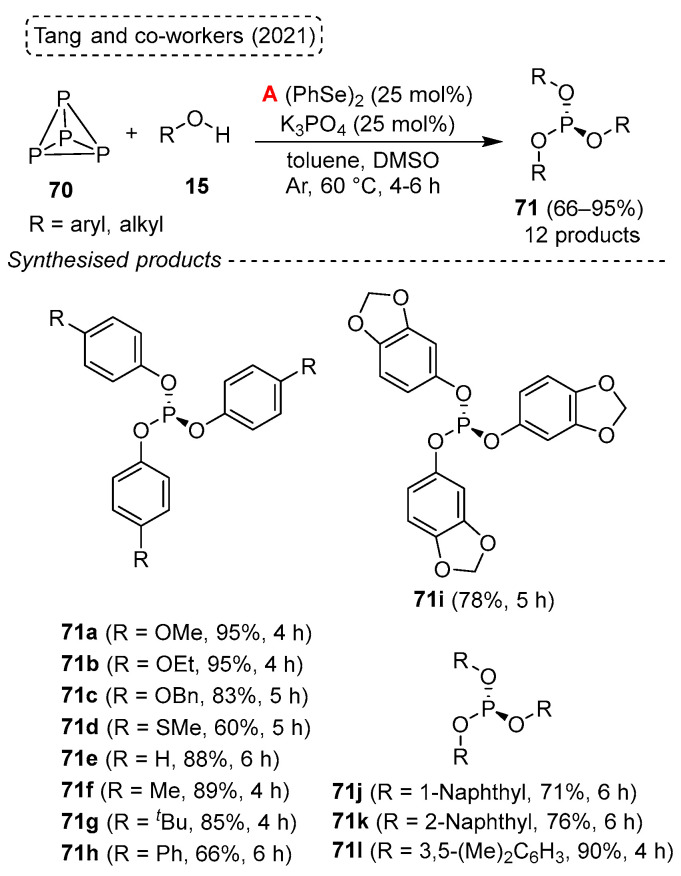
Synthesis of triaryl phosphite **71** [58].

In 2021, Tang and co-workers [58] described a halogen- and transition metal-free protocol for the synthesis of triaryl phosphite **71** through the reaction of phosphorus (P_4_) **70** with phenol **15** using diphenyl diselenide **A** as the catalyst. In this strategy, selenium catalyst **A** reacted with P_4_ to obtain the P–Se compound intermediate, which undergoes a nucleophilic substitution with phenol **15** to form the target triaryl phosphite **71** (Scheme 75).

Tang’s protocol tolerated several of the phenols of **15** when reacted with P_4_ (in a toluene solution), 25 mol% of K_3_PO_4_ as the base, 25 mol% of diphenyl diselenide **A** as the catalyst, and dimethyl sulfoxide (DMSO) as the solvent at 60 °C for 4–6 h under an argon atmosphere. Under these conditions, the target triaryl phosphites **71a**–**l** (12 compounds) were obtained, with yields ranging from good to excellent (66–95%). The method was efficient for several EDGs (Me, *^t^*Bu, MeO, EtO, BnO, or MeS) bonded in the aromatic ring of phenol **15**. Similar results were obtained for 1-naphthalenol, 2-naphthalenol, and 4-phenyl phenol; in these cases, the desired triaryl phosphites **71j**, **71k**, and **71h** were obtained at yields of 71% (6 h), 76% (6 h), and 66% (6 h), respectively. Phenol, as well as the phenol substituted with an acetal group, was also used as a starting material, which produced compounds **71e** and **71i** at yields of 88% (6 h) and 78% (5 h), respectively (Scheme 75).

**Scheme 76:**
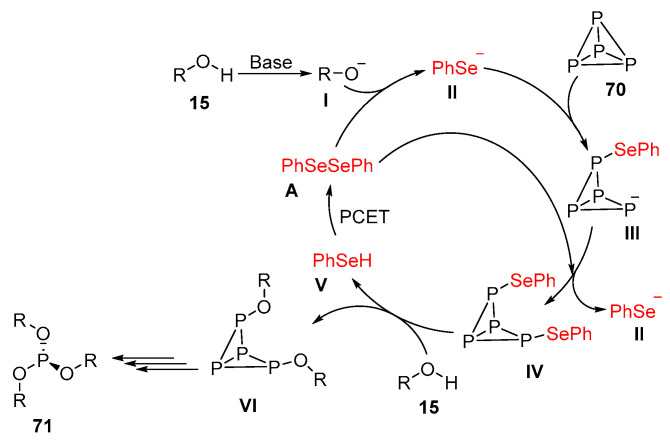
Proposed mechanism for the synthesis of triaryl phosphite **71**.

The proposed mechanism for the synthesis of triaryl phosphite **71** starts with the reaction of phenolates **I** (or a base) with diphenyl diselenide **A** to form anion PhSe^−^ **II**, which yields the intermediate **III** after a reaction with P_4_ **70**. Intermediate **III** reacts with diphenyl diselenide **A** to yield intermediate **IV** and regenerates anion **II**. Intermediate **IV** (previously formed) undergoes a nucleophilic attack from phenol derivative **15** to obtain intermediate **VI** and benzeneselenol **V**, which regenerate diphenyl diselenide **A** by proton-coupled electron transfer (PCET). The target triaryl phosphite, **71**, is formed after successive reactions with intermediate **VI** (Scheme 76).

## 3. Conclusions

The expansive number of papers discussed in this review reflects the interest in the use of diorganyl diselenides as catalysts. As described here, a range of several organic compounds with molecular diversity and high complexity were prepared at good to excellent yields. Diselenide shows chemical versatility, as reported in relation to the mechanisms; sometimes, diselenide is combined with fluor compounds or other oxidants, as well as LED irradiation and electrochemical conditions, among others. Interestingly, some diselenides can promote regio-, diastereo- and enantioselective reactions, highlighting the importance of their studies even more. Still, considerable efforts and improvements must be conducted in this field, targeting new catalysts based on diselenides allied with their new organic transformative applications. In this sense, we hope that this review encourages the synthetic chemistry community to develop further studies regarding the role of diselenide as a catalyst in these reactions by looking for a proven mechanism, as well as new conditions to promote the reaction with high efficiency.
